# Mechanism of Bazhen decoction in the treatment of colorectal cancer based on network pharmacology, molecular docking, and experimental validation

**DOI:** 10.3389/fimmu.2023.1235575

**Published:** 2023-09-20

**Authors:** Shuai Lu, Xibo Sun, Zhongbao Zhou, Huazhen Tang, Ruixue Xiao, Qingchen Lv, Bing Wang, Jinxiu Qu, Jinxuan Yu, Fang Sun, Zhuoya Deng, Yuying Tian, Cong Li, Zhenpeng Yang, Penghui Yang, Benqiang Rao

**Affiliations:** ^1^ Key Laboratory of Cancer Foods for Special Medical Purpose (FSMP) for State Market Regulation, Department of Gastrointestinal Surgery/Clinical Nutrition, Beijing Shijitan Hospital, Capital Medical University, Beijing International Science and Technology Cooperation Base for Cancer Metabolism and Nutrition, Beijing, China; ^2^ Department of Breast Surgery, The Second Affiliated Hospital of Shandong First Medical University, Shandong, China; ^3^ Department of Urology, Beijing TianTan Hospital, Capital Medical University, Beijing, China; ^4^ Key Laboratory of Molecular Pathology, Inner Mongolia Medical University, Hohhot, China; ^5^ Medical Laboratory College, Hebei North University, Zhangjiakou, China; ^6^ First Clinical Medical College, Binzhou Medical University, Yantai, China; ^7^ Institute of Hepatobiliary Surgery, The First Medical Center of Chinese People's Liberation Army (PLA) General Hospital, Beijing, China; ^8^ Department of General Surgery, Qilu Hospital of Shandong University, Jinan, China

**Keywords:** Bazhen detection, colored cancer, network pharmacology, molecular docking, tumor immunity

## Abstract

**Objective:**

Bazhen Decoction (BZD) is a common adjuvant therapy drug for colorectal cancer (CRC), although its anti-tumor mechanism is unknown. This study aims to explore the core components, key targets, and potential mechanisms of BZD treatment for CRC.

**Methods:**

The Traditional Chinese Medicine Systems Pharmacology (TCMSP) was employed to acquire the BZD’s active ingredient and targets. Meanwhile, the Drugbank, Therapeutic Target Database (TTD), DisGeNET, and GeneCards databases were used to retrieve pertinent targets for CRC. The Venn plot was used to obtain intersection targets. Cytoscape software was used to construct an “herb-ingredient-target” network and identify core targets. GO and KEGG pathway enrichment analyses were conducted using R language software. Molecular docking of key ingredients and core targets of drugs was accomplished using PyMol and Autodock Vina software. Cell and animal research confirmed Bazhen Decoction efficacy and mechanism in treating colorectal cancer.

**Results:**

BZD comprises 173 effective active ingredients. Using four databases, 761 targets related to CRC were identified. The intersection of BZD and CRC yielded 98 targets, which were utilized to construct the “herb-ingredient-target” network. The four key effector components with the most targets were quercetin, kaempferol, licochalcone A, and naringenin. Protein-protein interaction (PPI) analysis revealed that the core targets of BZD in treating CRC were AKT1, MYC, CASP3, ESR1, EGFR, HIF-1A, VEGFR, JUN, INS, and STAT3. The findings from molecular docking suggest that the core ingredient exhibits favorable binding potential with the core target. Furthermore, the GO and KEGG enrichment analysis demonstrates that BZD can modulate multiple signaling pathways related to CRC, like the T cell receptor, PI3K-Akt, apoptosis, P53, and VEGF signaling pathway. *In vitro*, studies have shown that BZD dose-dependently inhibits colon cancer cell growth and invasion and promotes apoptosis. Animal experiments have shown that BZD treatment can reverse abnormal expression of PI3K, AKT, MYC, EGFR, HIF-1A, VEGFR, JUN, STAT3, CASP3, and TP53 genes. BZD also increases the ratio of CD4^+^ T cells to CD8^+^ T cells in the spleen and tumor tissues, boosting IFN-γ expression, essential for anti-tumor immunity. Furthermore, BZD has the potential to downregulate the PD-1 expression on T cell surfaces, indicating its ability to effectively restore T cell function by inhibiting immune checkpoints. The results of HE staining suggest that BZD exhibits favorable safety profiles.

**Conclusion:**

BZD treats CRC through multiple components, targets, and metabolic pathways. BZD can reverse the abnormal expression of genes such as PI3K, AKT, MYC, EGFR, HIF-1A, VEGFR, JUN, STAT3, CASP3, and TP53, and suppresses the progression of colorectal cancer by regulating signaling pathways such as PI3K-AKT, P53, and VEGF. Furthermore, BZD can increase the number of T cells and promote T cell activation in tumor-bearing mice, enhancing the immune function against colorectal cancer. Among them, quercetin, kaempferol, licochalcone A, naringenin, and formaronetin are more highly predictive components related to the T cell activation in colorectal cancer mice. This study is of great significance for the development of novel anti-cancer drugs. It highlights the importance of network pharmacology-based approaches in studying complex traditional Chinese medicine formulations.

## 1 Introduction

CRC is the third most common malignant tumor and the second leading cause of cancer deaths worldwide. It accounts for 10% of cancer incidence and 9% of deaths (1). Despite the steady advancements in screening, diagnosis, and treatment of CRC in recent years (2–4), the CRC patients’ prognosis remains bleak due to the absence of early detection, frequent metastasis, and recurrence. CRC remains a global health issue. Traditional Chinese medicine, together with surgery, chemotherapy, radiotherapy, immunotherapy, and targeted therapy, can treat colorectal cancer, according to a recent pharmacological study. It has been found to effectively impede cancer progression and enhance the quality of life of cancer patients (5–7). As a valuable and rich source for advancing modern pharmacology, traditional Chinese medicine plays a distinctive role in mitigating adverse reactions in tumor treatment, reducing the likelihood of recurrence, and enhancing patients’ quality of life (8, 9). Traditional Chinese medicine has several advantages in treating tumors, like multi-ingredient, multi-target, and low drug resistance. Additionally, it has demonstrated promising outcomes in treating CRC (10). Due to its multi-ingredient and multi-target nature, traditional Chinese medicine has complex interactions across its targets, resulting in confusing molecular mechanisms and a gap between basic research and therapeutic applications. Consequently, addressing this issue has emerged as a pressing imperative for advancing traditional Chinese medicine.

Network pharmacology integrates bioinformatics, systems biology, and pharmacology to reveal the complex relationship between traditional Chinese medicine and disorders. It also follows the holistic and comprehensive concepts of Traditional Chinese Medicine (11, 12). Network pharmacology has updated the “one target, one drug” model to the “multi-component, multi-target” model, elucidating complex interactions between drugs and disease-related targets from a network perspective, providing a possibility for us to systematically study the relationship between traditional Chinese medicine and diseases (13, 14). Molecular docking simulates atomic-level interactions between small molecule compounds and protein targets, predicts ligand and receptor conformations, and calculates affinity to evaluate the combination. This technology is both low-cost and accurate and is primarily employed for drug design and elucidation of biochemical pathways (15). In recent years, network pharmacology and molecular docking technology have been extensively researched to identify active compounds and mechanisms of action in Chinese medicine (16, 17).

Traditional Chinese medicine considers the “spleen” an essential organ that digests and absorbs food. According to modern medicine, the stomach, small intestine, large intestine, and pancreas depend on the spleen. Traditional Chinese medicine theory also posits that each internal organ possesses its unique “qi,” with the spleen serving as the primary “qi” generation source. Qi is a fundamental substance that sustains the vital functions of the human body, augmenting immune defense and physiological processes. Spleen weakness reduces qi production, which impairs digestion and gastrointestinal tract immunity. Unhealthy dietary practices can generate “dampness toxins” that impair the spleen, resulting in reduced gas production, compromised intestinal immune function, and eventual cancer development (18). Hence, CRC pathogenesis is primarily attributed to spleen deficiency and qi deficiency. BZD, derived from the “Experience Formula of Rui Zhu Decoction,” is a classic formula with more than 700 years of history. It includes ginseng, Atractylodes macrocephala, Poria cocos, Angelica sinensis, Chuanxiong, Paeonia lactiflora, Rehmannia glutinosa, and licorice, and is known for its ability to nourish qi and blood. Qi and blood deficit caused by a post-disease imbalance or excessive blood loss are treated with it. According to a study, BZD improves immune function and bone marrow hematopoietic function 0 (19, 20). Recently, it has been extensively employed in immunizing diverse malignant tumors and as adjuvant therapy for radiotherapy and chemotherapy, resulting in favorable clinical outcomes. BZD has the potential to augment the immune function of cancer patients (21–23), mitigate the toxic side effects of chemotherapy drugs (24, 25), and enhance patient prognosis (26, 27). BZD treats several cancers, including CRC, gastric lung, breast, cervical cancer, and acute lymphoblastic leukemia (27–32). Xu et al.’s clinical investigations have demonstrated that the combination of BZD and capecitabine, as opposed to capecitabine monotherapy, can decrease the likelihood of disease progression in elderly patients with advanced CRC, provide superior survival advantages, and reduce the incidence of chemotherapy-related adverse reactions, thereby significantly facilitating patients’ fatigue and gastrointestinal symptoms (33). Zhou et al. also revealed that combining BZD and chemotherapy can improve advanced colon cancer treatment and survival outcomes (34). BZD’s mechanism in treating CRC is unknown. A better understanding of the regulatory role of herbs in cancer will provide new avenues for cancer treatment.

**Figure 1:**
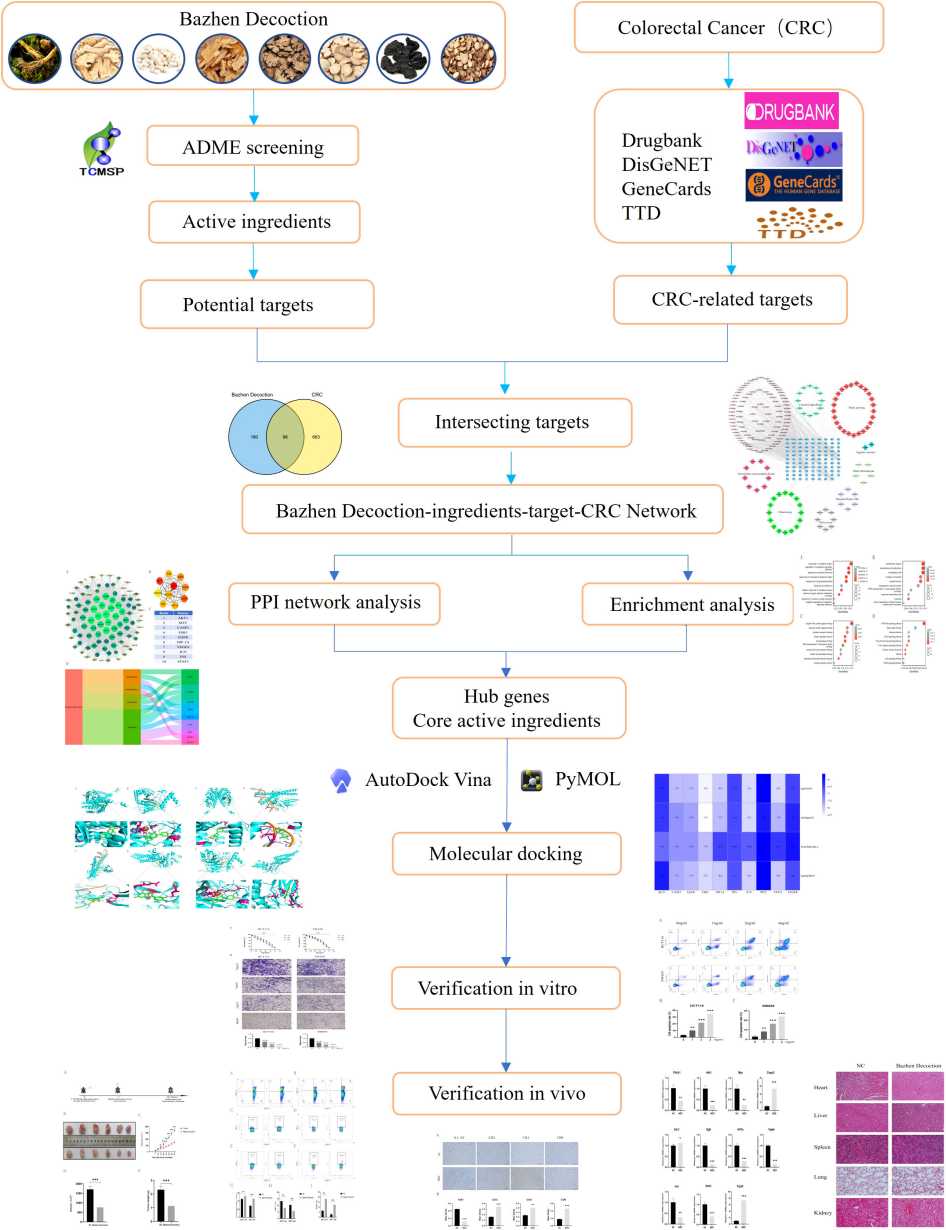
The flowchart of this study.

This study employed network pharmacology study examined BZD’s active components, targets, and mechanism of action in CRC treatment. The predictions were subsequently validated through molecular docking and *in vitro* and *in vivo* experimental studies. Additionally, a target network was established to elucidate the interaction between drug ingredients and diseases, thereby providing a foundation for comprehending the mechanism of action of BZD in treating CRC. The study’s methodology is depicted in **Figure 1**.

## 2 Materials and methods

### 2.1 Collection of effective ingredients and targets of BZD

The TCMSP database (https://tcmspw.com/tcmsp.php) was used to find the chemical components of various drugs in BZD. This study selected the criteria of oral bioavailability (OB) ≥ 20% and drug-likeness (DL) ≥ 0.1 as effective ingredient screening conditions (35). The active drugs’ target proteins were matched with TCMID and DrugBank databases, and then these target proteins were standardized to human species genes through the UniProt database.

### 2.2 Collection of CRC targets

The targets for colorectal cancer (CRC) were obtained from four databases, namely Drugbank (https://go.drugbank.com/) (36), GeneCards (https://genecards.weizmann.ac.il/v3/) (37), TTD (https://db.idrblab.net/ttd/) (38), and Dis-GeNET (https://www.disgenet.org/) (39). The search results from these databases were integrated, and any duplicate targets were eliminated.

### 2.3 Intersection target and construction of “herb-ingredient-target” network

The BZD and CRC intersection target was obtained using R language version 4.2.1. Integrate herbs, active ingredients, and intersection targets into Cytoscape 3.8.0 to construct a “herb- ingredient-target” network (40).

### 2.4 Analysis of protein-protein interaction

Intersection target of BZD and CRC input to STRING database (https://string-db.org/), select protein interaction data with a confidence level (score>0.4), and save it in a TSV format file. The information of node1, node2, and combination scores was imported from the file into Cytoscape software to construct a PPI network and screened hub genes through the cytohub plugin. The R language version 4.2.1 be used to draw Sangi diagrams of drugs, core components, and central genes.

### 2.5 GO and KEGG enrichment analysis

The “ClusterProfiler” package was employed to conduct GO and KEGG enrichment analysis on intersecting targets. The visualization of the enrichment analysis outcomes was accomplished by the “ggplot2” package, while the R language version 4.2.1 was utilized to generate the bubble chart.

### 2.6 Molecular docking

The key ingredient’s 3D structural files in SDF format were obtained from the PubChem database and converted to PDB format using Open Babel. The 3D crystal structure of the hub gene was also obtained from the PDB database (https://www.pdbus.org/) and processed by removing ions and water molecules through PyMol 2.4.0 (41), resulting in PDB files. The essential ingredients and core targets were translated to PDBQT format to find the active pocket. Finally, molecular docking simulation was conducted using Autodock Vina software and visualized using PyMol 2.4.0.

### 2.7 Preparation of BZD freeze-dried powder

BZD comprises ginseng, atractylodes macrocephala, poria cocos, licorice, chuanxiong, paeonia lactiflora, rehmannia glutinosa, and angelica sinensis (Tongren Tang Pharmacy, Beijing, China) at a dose ratio of 1:1:1:1:1:1:1:1 as per the Orthodox Tradition of Medicine. Production method: We placed the above medicinal herbs in pure water, covered the top of the herbs with pure water, soaked them for 1 h, boiled them for 40 min, and then poured out the liquid. Then we added pure water, which still needed to cover the top of the herb, and boiled directly. After 30 min boiling, we poured out the liquid. Then two types of water extracts were mixed and filtered with gauze. The supernatant was concentrated to 300 mL by a rotary evaporator. Freeze the supernatant overnight at -80°C. The freeze-dried product was freeze-dried to produce 51g of powder. In *in vitro* experiments, we dissolved BZD powder in the required concentration of culture medium. The negative control used a culture medium without BZD. In animal experiments, BZD was administered orally to mice.

### 2.8 Cell culture

The CRC cell lines, namely HCT116, SW620, and MC38, were procured from Procell Life Science&Technology Co., Ltd. (Procell, Wuhan, China). The SW620 and MC38 cell lines were cultured in DMEM medium (Gibco, USA) enhanced with 10% fetal bovine serum (FBS) and 1% antibiotics (100 U/mL penicillin and 100 μG/mL streptomycin). HCT116 was maintained in RPMI-1640 medium supplemented with 10% FBS (Gibco, USA) and 1% penicillin/streptomycin (Gibco).

### 2.9 Cell proliferation detection

The BZD’s inhibitory effect on human CRC cells was assessed using CCK-8 (Dojindo, Kumamoto, Japan) reagent. 5000 HCT116 and SW620 cells were seeded per well in a 96-well plate. The cells are treated with BZD or cell culture medium without BZD for 12, 24, or 48 hours after adhering to the wall. Following treatment, the tumor cells were washed twice with PBS and incubated with a 1:10 diluted CCK-8 reagent in a serum-free medium. After a 2-h incubation at 37 °C, 100 uL of the diluted CCK-8 reagent was added to each well of the cell culture plate. The absorbance of the cells at 450 nm was observed at three experimental nodes.

### 2.10 Cell invasion detection

Cell invasion experiments were performed utilizing a 24-well Transwell plate (Corning, USA). HCT116 and SW620 cells were added to the Transwell chamber with a pore size of 0.8 μm (BioCoat, 354480), and the invasiveness of the cells was assessed. Briefly, 600 μL of complete culture media with 10% FBS was applied to each lower chamber well of the 24-well plate. Subsequently, 2 × 10^5^ cells were treated with 100 μL of resuspended serum-free culture medium containing varying BZD concentrations (0, 1, 2, and 4 mg/mL). They were then inoculated in the upper chamber and cultured in a cell incubator. After 24 h of cultivation, gently wipe the non-metastatic cells in the upper compartment with a damp cotton swab. Fixed the cells invading the lower lumen with a 4% paraformaldehyde solution for 30 min and then stained with a 0.1% crystal violet solution for 20 min. Subsequently, photos were taken using a microscope and statistically analyzed.

### 2.11 Cell apoptosis detection

BZD’s effect on colorectal cancer cell apoptosis was examined using flow cytometry. Logarithmic growth stage HCT116 and SW620 cells were seeded in a 6-well plate at a density of 2 × 10^5^ cells per well. Following cell adhesion, varying concentrations of Bazhen decoction (0, 1, 2, and 4 mg/mL) were administered for intervention. After 24 h, cells were harvested following the instructions of the membrane-associated protein V-APC/7-AAD cell apoptosis kit (Elabscience, E-CK-A218) and analyzed by flow cytometry within 30 min.

### 2.12 Animal experiments

Female C57BL/6 mice aged 4–6 weeks were obtained from SPF (Beijing) Biotechnology Co., Ltd. and maintained in an SPF environment. Following a week of adaptive feeding, subcutaneously injected 100 μL of MC38 cell suspension with 1 × 10^6^ cells into the right side of each mouse. The experiment commenced on the 7th day after tumor inoculation, when the tumor volume reached approximately 100 mm^3^. The mice were randomly allocated into two groups: the control group received daily gavage of sterile water (200 µL), and the BZD group received daily BZD (200 µL, 6.63 g/kg) for three weeks. The daily dose of BZD was determined based on the average adult body weight of 70 kg and a conversion coefficient 9.1 between humans and mice (42, 43). If the clinical drug dose for a 70 kg adult is X mg/kg, then the dosage for a 20 g mouse would be X mg/kg × 70 kg/0.02 kg × 0.0026. Since the clinical use of BZD for adults is 1 pair/day (51 g freeze-dried powder), the drug dose for a 70 kg adult would be about 728.6 mg/kg. By the above formula, the daily dosage for mice can be calculated as 6.63 g/kg, and the dosage of BZD for a 20 g mouse is 132.6 mg per day, with a gavage volume of 0.2 mL. Therefore, 663 mg/mL of sterile water was prepared to administer BZD. The tumor size was measured every two days using a digital caliper, and the tumor volume was calculated using the formula: tumor volume (mm^3^) = length × width × width × width × 0.52. Kaplan Meier survival curves were plotted after three weeks. The mice were euthanized, and their heart, liver, spleen, lungs, kidneys, and tumor tissues were collected for study. All experimental procedures were authorized by the Animal Ethics Committee of Beijing Shijitan Hospital, Affiliated with Capital Medical University (The ethical approval permit numbers are SJTKY11-1X-2021(59)).

### 2.13 Quantitative real-time polymerase chain reaction

The total RNA was extracted from the mice tumor tissues in the control and the Ba Zhen Decoction group using TRIzol reagent. The RNA was then converted into cDNA using TransScript first-strand cDNA synthesis SuperMix (TransGen Biotech, AT301). Then, qRT-PCR was performed using SYBR Green Master Mix (Applied Biosystems, USA). All primers employed for PCR amplification were designed using the NCBI Primer-BLAST and bought from Beijing Liuhe Huada Gene Technology Co., Ltd. The primer sequences are shown in **Table 1**. The GAPDH gene expression was determined simultaneously as an internal control. The relative gene expression was determined using the 2-ΔΔCT method. All samples were run in triplicate. Each primer pair’s specificity was validated by computer analysis (NCBI primer BLAST) and melt curve analysis after qPCR amplification.

**Table 1:** Sequences of PCR primers.

Gene symbol	Accession number	Forward primer (5′–3′)	Reverse primer (5′–3′)	Amplicon size
Gapdh	NM_001289726.2	AGGTCGGTGTGAACGGATTTG	TGTAGACCATGTAGTTGAGGTCA	147
Pik3r1	NM_001024955.2	TGGACTATGGAAGACCTGGACTTAGAG	TTGTTGTTCATGCTGTTGTTGGCTAC	149
Akt1	NM_001165894.2	ATGAACGACGTAGCCATTGTG	TTGTAGCCAATAAAGGTGCCAT	116
Myc	NM_001177352.1	CCCTATTTCATCTGCGACGAG	GAGAAGGACGTAGCGACCG	185
Casp3	NM_001284409.1	ATGGAGAACAACAAAACCTCAGT	TTGCTCCCATGTATGGTCTTTAC	74
Esr1	NM_001302531.1	CCCGCCTTCTACAGGTCTAAT	CTTTCTCGTTACTGCTGGACAG	76
Egfr	NM_007912.4	GCCATCTGGGCCAAAGATACC	GTCTTCGCATGAATAGGCCAAT	101
Hif1a	NM_001313919.2	CCACAACTGCCACCACTGATGAA	TGCCACTGTATGCTGATGCCTTAG	138
Vegfa	NM_001025250.3	GCACATAGAGAGAATGAGCTTCC	CTCCGCTCTGAACAAGGCT	105
Jun	NM_010591.2	TTCCTCCAGTCCGAGAGCG	TGAGAAGGTCCGAGTTCTTGG	133
Stat3	NM_011486.5	GCTTGGGCATCAATCCTGTGGTAT	GCTTGGTGGTGGACGAGAACTG	136
Trp53	NM_001127233.1	CACAGCACATGACGGAGGTC	TCCTTCCACCCGGATAAGATG	101

### 2.14 T lymphocyte activation

To analyze T cell activation and immune cell phenotype, the spleen of MC38 tumor-bearing mice was taken, minced, and ground in a 40-µm cell strainer. Then, the single-cell suspension was collected by filling it with staining buffer (PBS containing 3% FBS). Cell surface staining was performed by staining the single-cell suspension with APC-Cy7 anti-mouse CD45, FITC anti-mouse CD3, PerCP-Cy5.5 anti-mouse CD4, PE-Cy7 anti-mouse CD8, and BV421 anti-mouse PD-1 antibodies at room temperature for 30 minutes, followed by detection using a flow cytometer. For intracellular staining of the T cell cytokine IFN-γ, the filtered cell suspension was stimulated with Cell Activation Cocktail (BioLegend, 423303) for 6 h. Then the cells were collected and stained for surface markers (44–48). Fixation Buffer (BioLegend, 420801) fixed the cells at room temperature for 20–30 min. After two washes with 1X Permeabilization Buffer (BioLegend, 421002), the cells were stained with APC anti-mouse IFN- γ for 20 min. After washing the samples with a staining buffer, they were detected using a flow cytometer. The data were analyzed and visualized using FlowJo software.

### 2.15 Pathological and immunohistochemical testing

To assess the BZD’s safety, tissue samples from mice’s heart, liver, spleen, lungs, and kidneys were procured, embedded in paraffin, sectioned, and subjected to hematoxylin and eosin (H&E) staining. Additionally, tumor samples from each mice group were obtained, embedded in paraffin, sectioned, and subjected to immunohistochemical testing by CD3, CD4, CD8, and Ki67 antibodies. Image-Pro was used to calculate each picture’s integrated optical density (IOD) and area. Mean density (IOD/area) was used to analyze protein expression.

### 2.16 Statistical analysis

The statistical analysis of the data was performed using GraphPad Prism software V9.0. The t-tests were utilized to measure the differences between the two groups, while the one-way analysis of variance (ANOVA) was employed to evaluate the comparisons between the groups. The experimental data was presented as mean ± standard deviation, with a statistical significance level of p<0.05. The differences were denoted as ns, P > 0.05,* p ≤ 0.05, ** p ≤ 0.01, and *** p ≤ 0.001.

## 3 Results

### 3.1 Active ingredients screening

The TCMSP database provided the drug’s active components using ADME screening conditions. The database yielded 69 ginseng, 19 *atractylodes macrocephala*, 19 *poria cocos*, 11 *angelica sinensis*, 35 *ligusticum chuanxiong*, 28 *paeonia lactiflora*, 24 *rehmannia glutinosa*, and 126 *glycyrrhiza uralensis* ingredients. The database also provided 258 ingredient target genes.

### 3.2 CRC targets acquisition

**Figure 2:**
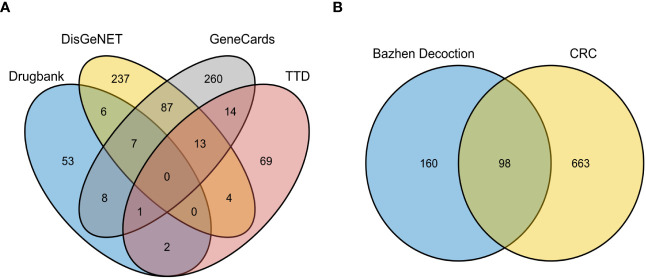
Intersection Target of CRC and Ingredient Action Target of BZD with CRC. **(A)** Venn diagram displaying CRC-related targets among the four databases. **(B)** Venn diagram of the intersection target of BZD and CRC.

Briefly, 104, 77, 390, and 353 targets were obtained from TTD, Drugbank, GeneCards, and DisGeNET databases, respectively. After merging and removing duplicates of disease targets from the four databases, 761 colorectal cancer-related targets were retained (**Figure 2A**).

### 3.3 “Herb-ingredient-target” network construction

**Figure 3:**
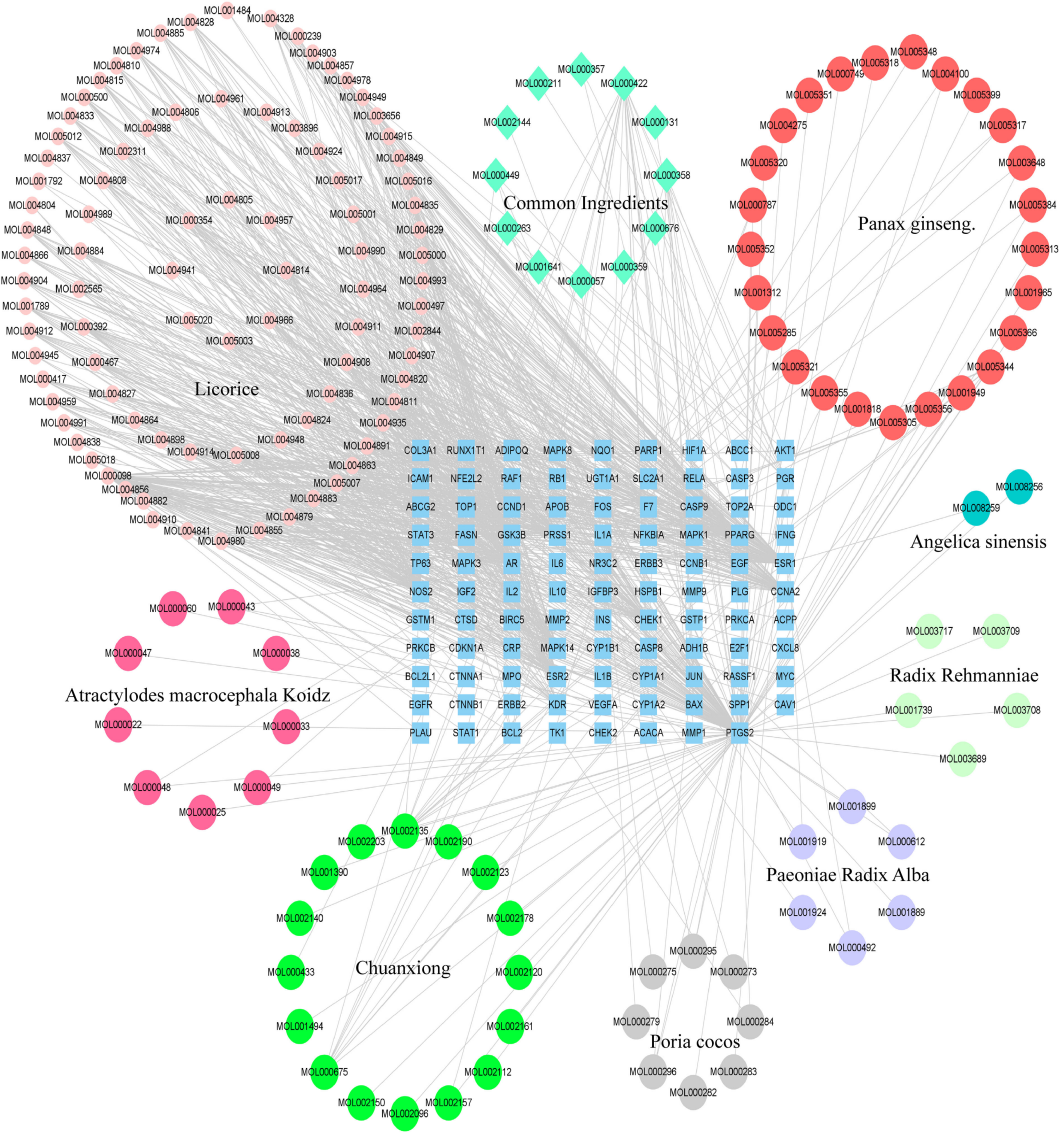
The “herbal-ingredient-target” network of BZD in treating CRC.

The BZD’s component action targets were intersected with targets correlated to colorectal cancer, identifying 98 intersecting targets (**Figure 2B**). Subsequently, 173 effective ingredients, 98 cross targets, and individual drug names that BZD can act on colorectal cancer targets were imported into Cytoscape 3.8.0 to construct a “herbal- ingredient -target” network (**Figure 3**).

Nodes with greater degree values may be essential in a network. Quercetin displays the highest number of targets, encompassing 73 potential targets, followed by kaempferol, licorice ketone, and naringin, with 22, 16, and 15 targets, respectively. BZD’s key ingredients may be these active compounds with more targets. **Table 2** presents the top 10 active ingredients in the degree ranking.

**Table 2:** The top ten ingredients in the herbal ingredient CRC target network.

Mol ID	Degree	Molecule name
MOL000098	73	quercetin
MOL000422	22	kaempferol
MOL000497	16	licochalcone A
MOL004328	15	naringenin
MOL000354	13	isorhamnetin
MOL004966	13	3’-Hydroxy-4’-O-Methylglabridin
MOL002135	12	Myricanone
MOL001789	12	isoliquiritigenin
MOL000392	12	formononetin
MOL004828	12	Glepidotin A

### 3.4 Protein-protein interaction analysis of intersection targets

**Figure 4:**
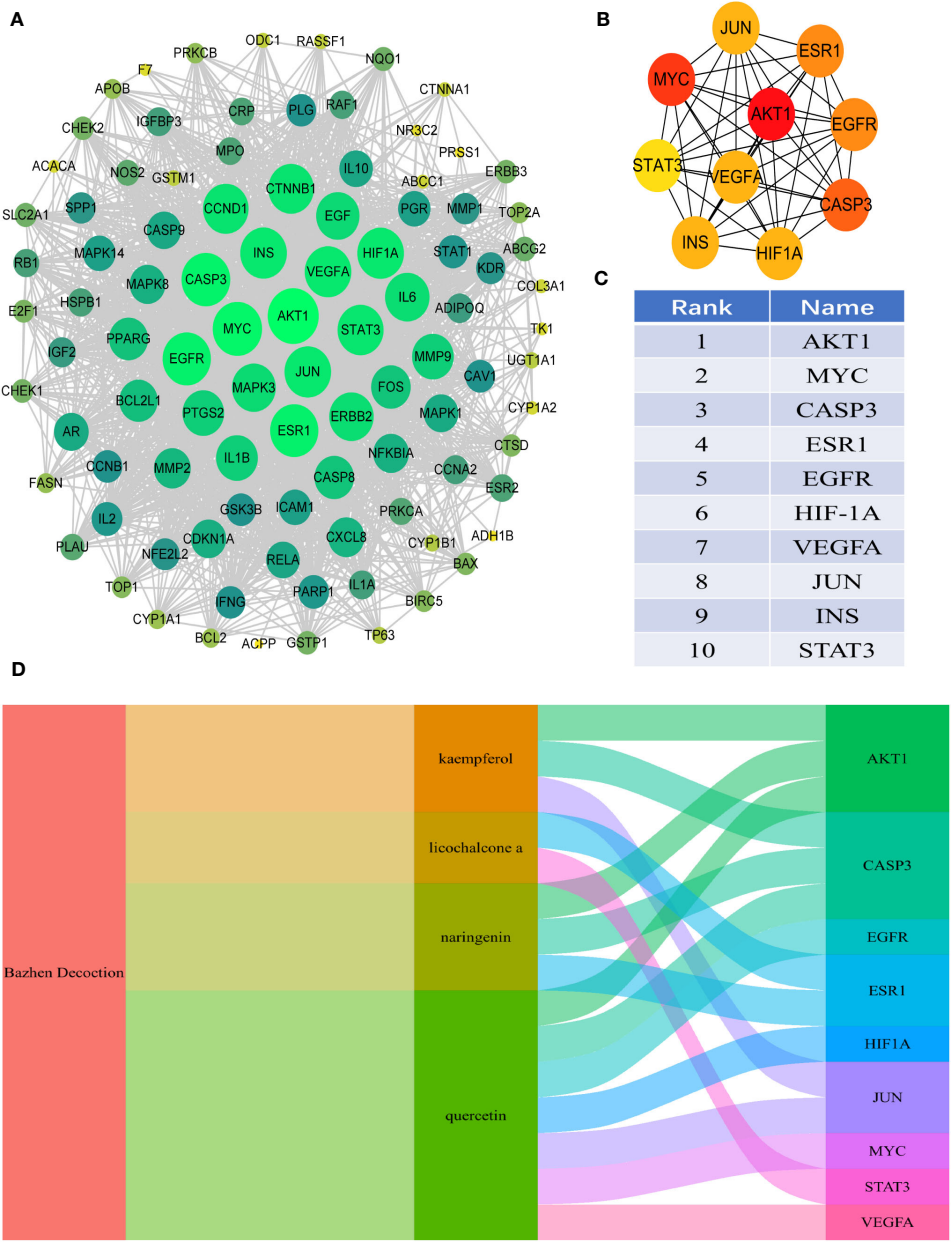
PPI analysis of the intersection target between BZD and CRC. **(A)** PPI interaction network between BZD and the intersection target of CRC. **(B, C)** The top 10 core genes were obtained from PPI network analysis. **(D)** The Sankey diagram reveals the relationship between BZD, core ingredients, and central genes.

Ninety-eight intersection targets of BZD and CRC were imported into the STRING 11.0 database (https://www.string-db.org/) for analysis, acquiring a PPI network of intersection targets between BZD and CRC. The network comprises 98 nodes and 1835 edges. Nodes indicate intersecting targets, while edges express associations between them. Cytoscape 3.8.0 was employed to present the PPI network diagram (**Figure 4A**), while the Cytohubba plugin was utilized to compute the target set for further refinement of the core targets. The top 10 hub genes in degree ranking, AKT1, MYC, CASP3, ESR1, EGFR, HIF1A, VEGFA, JUN, INS, and STAT3, were identified (**Figures 4B, C**). The Sankey diagram also showed the link between BZD, the four basic components, and the ten core genes (**Figure 4D**).

### 3.5 GO and KEGG enrichment analysis of intersecting targets

**Figure 5:**
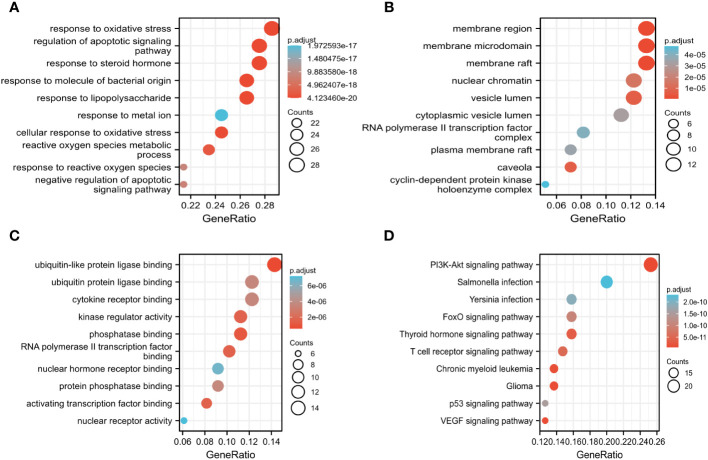
GO, and KEGG analysis determine the key biological processes of BZD in treating CRC. **(A–C)** GO enrichment analysis results in biological processes (BP), cellular ingredients (CC), and molecular functions (MF). **(D)** KEGG enrichment analysis results.

The key biological functions of BZD in treating CRC were determined by utilizing GO and KEGG enrichment analysis. The bubble plots of the top 10 biological processes (BP), cellular composition (CC), and molecular function (MF) in GO analysis are presented in **Figures 5A-C**. The biological processes focus on cell death signals and oxidative stress responses. In contrast, the cellular composition changes are mostly related to membrane rafts, membrane microregions, and cyclin-dependent protein kinase holoenzyme complexes. The MF modifications primarily focused on binding with ubiquitin-like protein ligase, RNA polymerase II transcription factor, ubiquitin protein connexin, and protein phosphatase. Through KEGG pathway enrichment analysis, 165 signal pathways were identified. The KEGG enrichment analysis indicates that the PI3K-AKT, T cell receptor, P53, and VEGF signaling pathways could potentially serve as crucial pathways for treating colorectal cancer with BZD, as illustrated in **Figure 5D**.

### 3.6 Molecular docking

**Figure 6:**
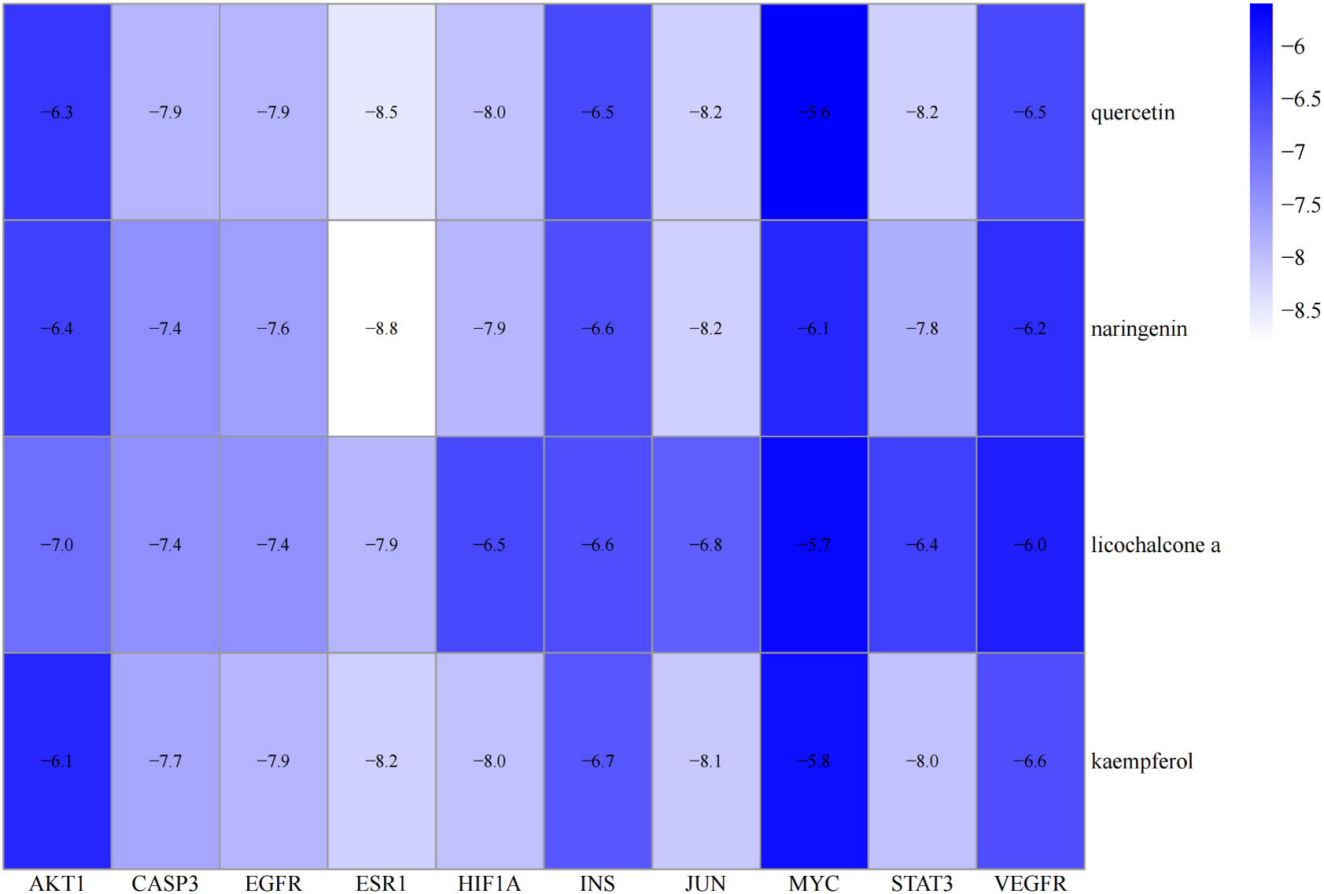
Heatmap of the binding energy of molecular docking between 4 core ingredients and 10 core targets.

Molecular docking was performed on four core ingredients (quercetin, naringenin, licochalcone A, kaempferol) and 10 hub genes (AKT1, CASP3, EGFR, ESR1, HIF1A, INS, JUN, MYC, STAT3, and VEGFR) to assess the protein-ligand binding potential. Affinity refers to the capacity of a ligand to bind with receptors, and a higher absolute affinity value indicates a stronger binding ability (with a negative value). **Figure 6** illustrates the binding energy of the core ingredients docked with the hub genes. The findings indicate that quercetin exhibits favorable docking effects with ESR1, HIF-1A, JUN, and STAT3, with respective binding energies of -8.5, -8.0, -8.2, and -8.2 kcal/mol. Naringenin’s binding energies of -8.8 and -8.2 kcal/mol with ESR1 and JUN are favorable.

**Figure 7:**
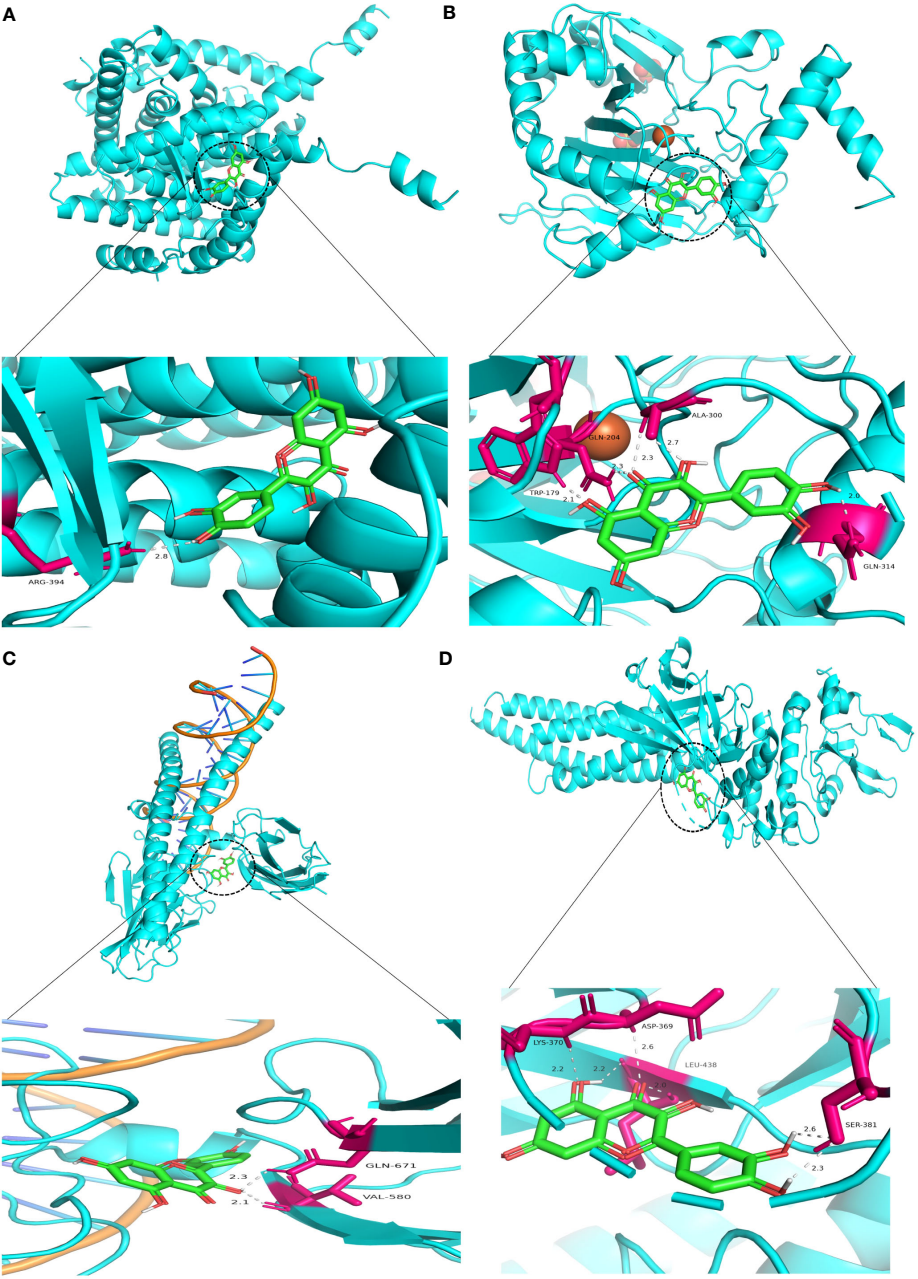
Molecular docking model 3D diagram. Quercetin binds to ESR1 **(A)**, HIF-1A **(B)**, JUN **(C)**, and STAT3 **(D)**.

**Figure 8:**
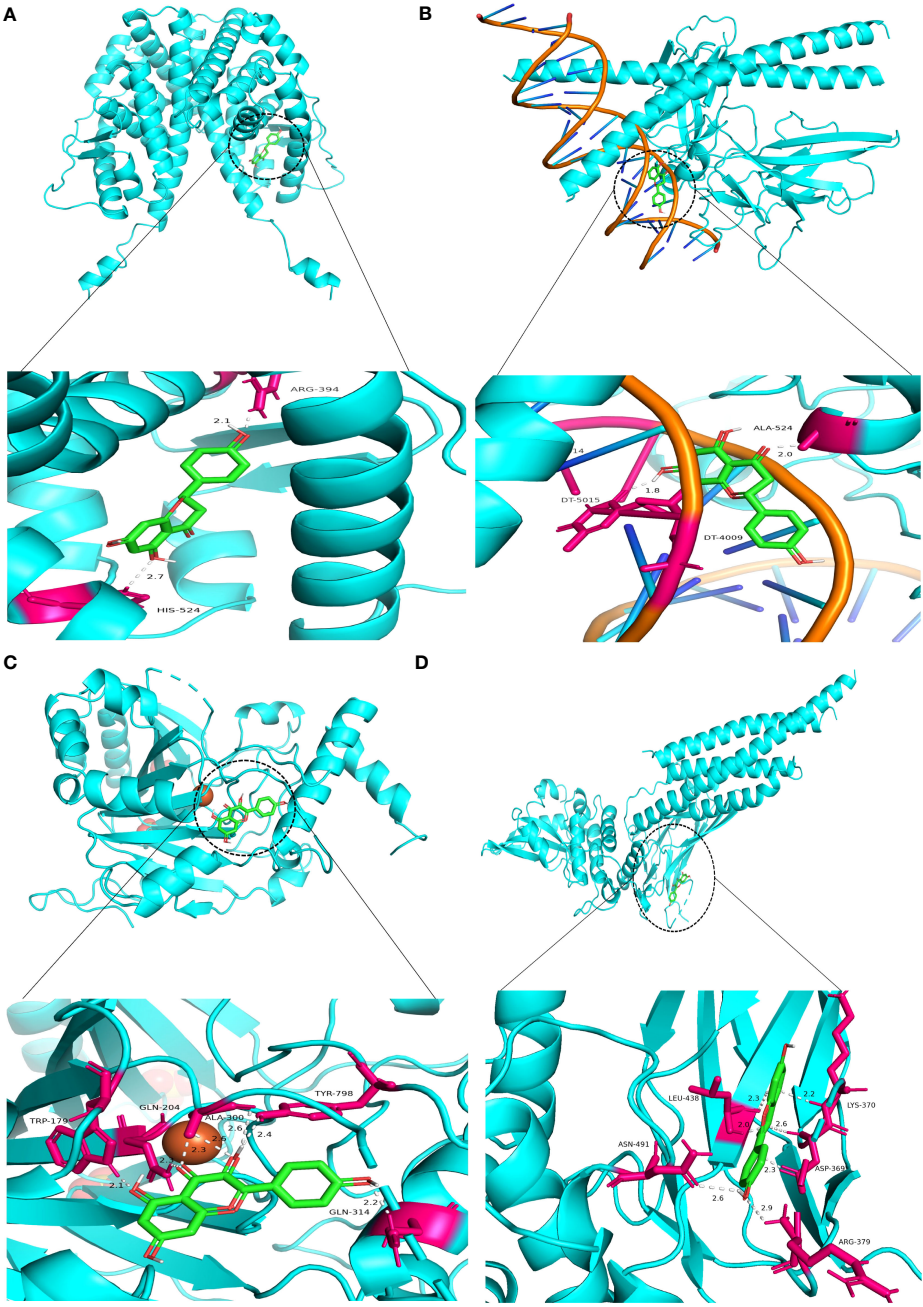
Molecular docking model 3D diagram. Naringin binds to ESR1 **(A)** and JUN **(B)**, while kaempferol binds to HIF-1A **(C)** and STAT3 **(D)**.

Similarly, Kaempferol demonstrates a favorable docking effect with HIF-1A, STAT3, ESR1, and JUN, with corresponding binding energies of -8.0 kcal/mol, -8.0 kcal/mol, -8.2 kcal/mol, and -8.1 kcal/mol, respectively. The molecular docking models are depicted in a 3D diagram using PyMol 2.4.0. **Figure 7** illustrates the interactions between quercetin and ESR1, HIF-1A, JUN, and STAT3. **Figure 8** showcases the interactions between naringin and ESR1, JUN, and the interaction between kaempferol and HIF-1A and STAT3.

### 3.7 In a time- and dose-dependent manner, BZD suppresses the proliferation and invasion of CRC cells while facilitating their apoptosis

**Figure 9:**
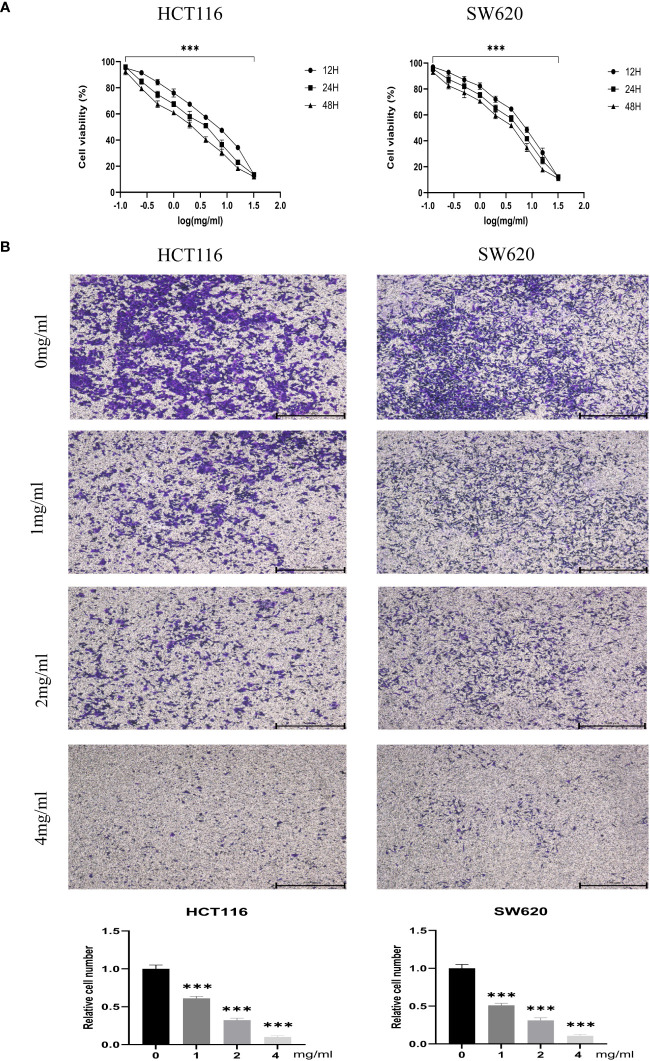
BZD inhibits the proliferation and invasion of colon cancer cells in a dose-dependent manner. **(A)** HCT116 and SW620 cells were subjected to 10 concentration gradients of BZD for 12, 24, and 48 h, and the resulting cell viability was assessed using CCK8. **(B)** The invasiveness of HCT116 and SW620 cells was measured after exposure to four BZD concentration gradients for 24 h. ***P < 0.001.

CCK-8 assays measured the proliferation of CRC cell lines HCT116 and SW620 treated with different BZD doses. Ten concentration gradients were used, which are 0, 0.125, 0.25, 0.5, 1, 2, 4, 8, 16, and 32 mg/mL. The findings indicate that BZD effectively suppressed the CRC cells’ activity in a dose- and time-dependent manner, as illustrated in **Figure 9A**.

**Figure 10:**
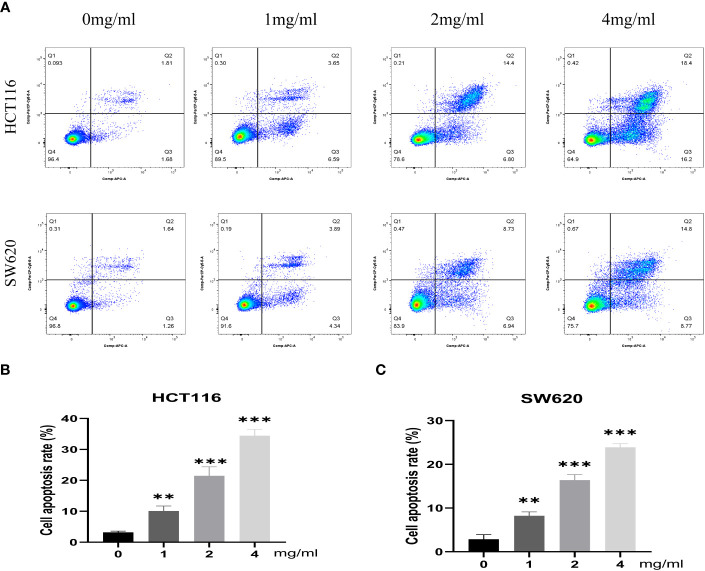
BZD promotes apoptosis of colon cancer cells in a dose-dependent manner. **(A)** Flow cytometry revealing that BZD can dose-dependently induce apoptosis in HCT116 and SW620 cells. **(B, C)** Histogram showing that BZD can induce apoptosis in HCT116 and SW620 cells in a dose-dependent manner. **P < 0.01, ***P < 0.001.

Furthermore, the impact of varying concentrations (0, 1, 2, 4 mg/mL) of BZD on the invasion capacity and apoptotic rate of the two CRC cell lines was assessed. The findings indicated that BZD exhibited a dose-dependent suppression of CRC cell invasion (**Figure 9B**) and promoted apoptosis of CRC cells (**Figure 10**).

### 3.8 BZD inhibits tumor progression in mice

**Figure 11:**
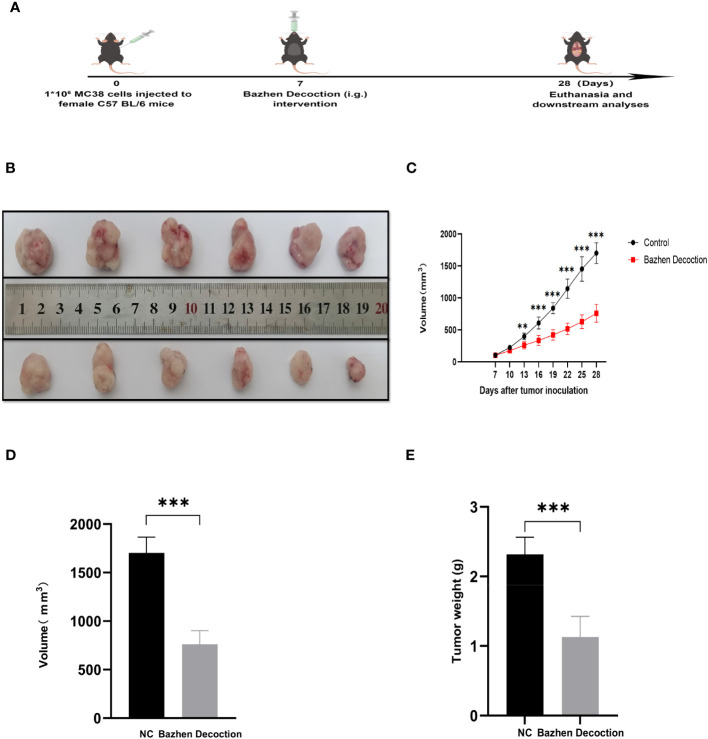
BZD inhibits tumor progression in MC38 tumor-bearing mice. **(A)** Schedule of MC38 tumor-bearing mouse model. **(B)** On the 28th day, images of tumors in both groups of mice were obtained. **(C)** Curve plots of tumor volume change in two groups of tumors bearing mice. **(D)** Histograms of tumor volume comparison between two groups of tumors bearing mice. **(E)** Histogram comparison of tumor weight between two groups of tumors bearing mice. **P < 0.01, ***P < 0.001.

**Figure 12:**
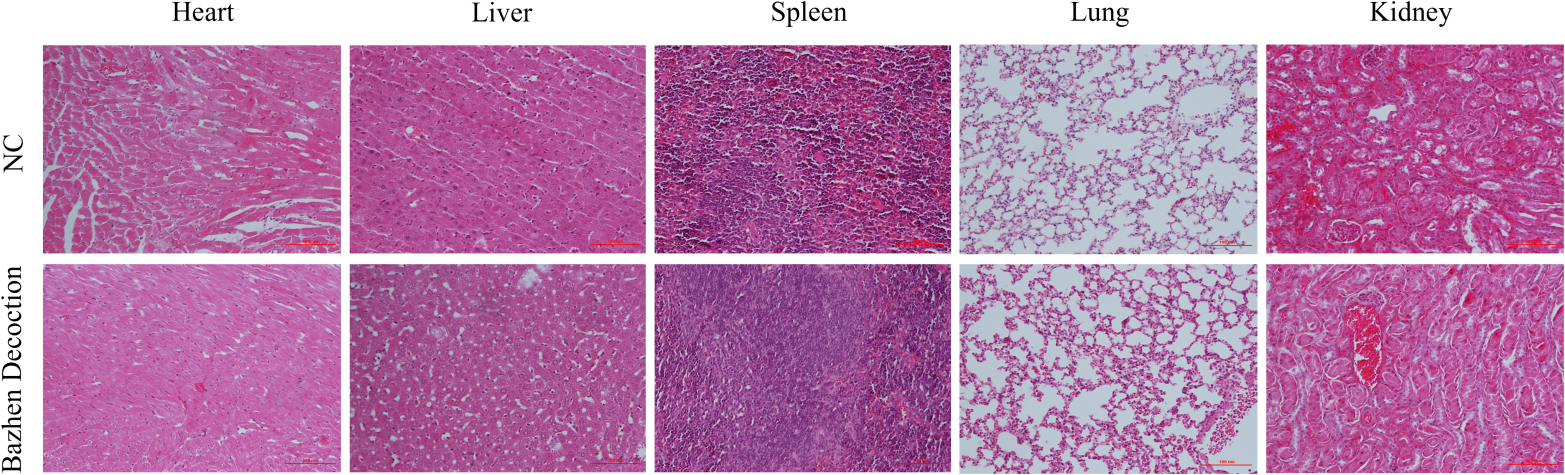
Organ toxicity of BZD. Two groups of mice were euthanized at the end of treatment, and their hearts, liver, spleen, lungs, and kidneys were removed for H&E staining to evaluate organ toxicity (scale: 100 mm).

An MC38 tumor-bearing mouse model was created to test BZD’s CRC treatment efficacy. **Figure 11A** shows the mice’s experimental protocol. Following three weeks of consistent oral administration of BZD, a noteworthy reduction in subcutaneous tumor volume was observed in the BZD group compared to the sterile water group (p ≤ 0.001; **Figure 11B**), as illustrated by the tumor volume curve in **Figure 11C**. After 21 days, the control group had a mean tumor size of 1703.15 mm^3^, while the BZD group had 760.94 mm^3^ (**Figure 11D**). On day 28, the mice were humanely euthanized to obtain the tumor. The average tumor weights for the control and BZD groups were 2.32 and 1.13 g, respectively (**Figure 11E**). H&E staining of the heart, liver, spleen, lungs, and kidneys showed no significant differences between experimental groups (**Figure 12**), demonstrating that BZD is non-toxic and does not damage tissue. Consequently, the BZD intragastric administration effectively suppressed the development of subcutaneous tumors in MC38 mice.

### 3.9 The effect of BZD on key genes and KEGG pathway-related genes

**Figure 13:**
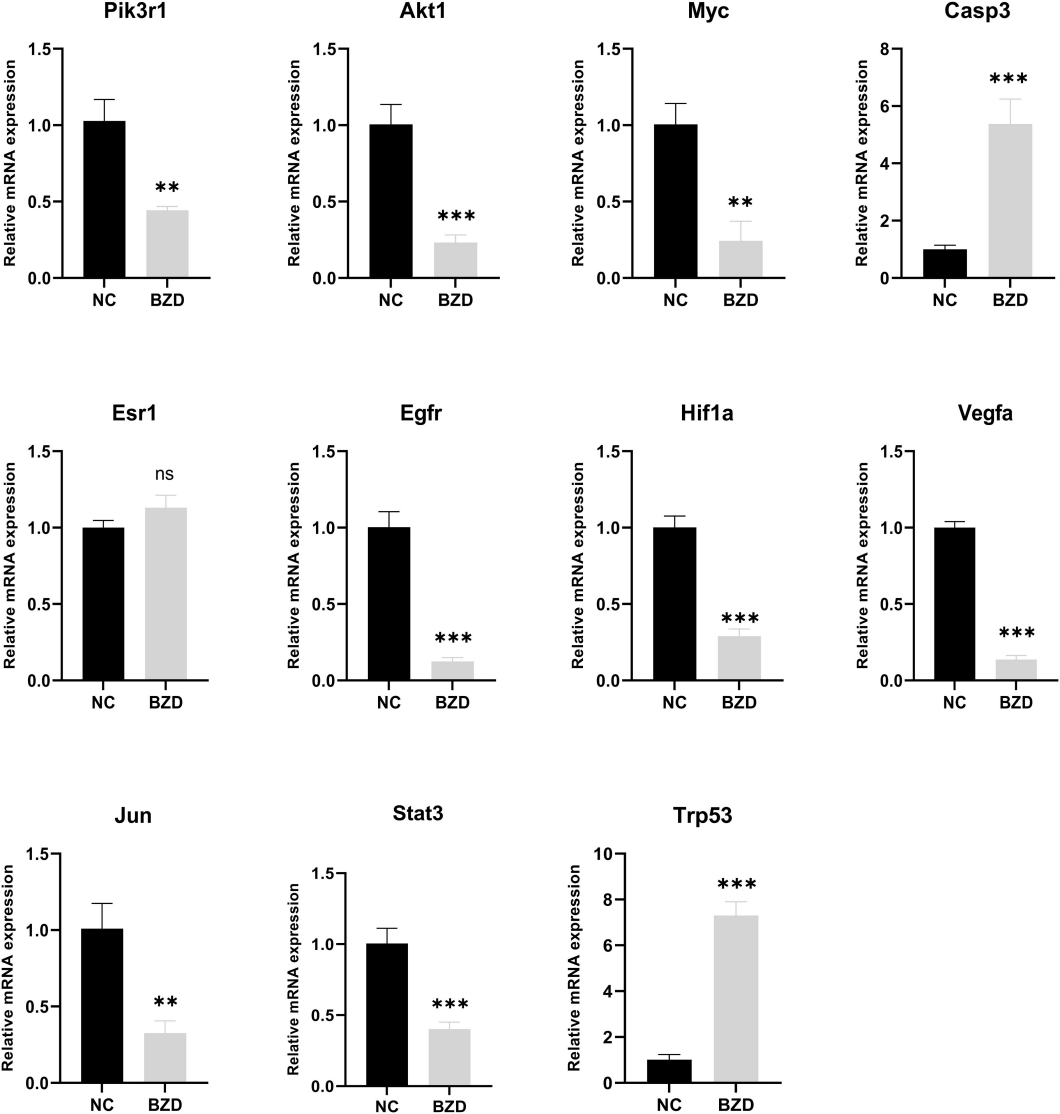
Effect of BZD on Key Genes and KEGG Pathway-Related Genes. Compared to NC group, mRNA expression levels of Pik3r1, Akt1, Myc, Esr1, Egfr, Hif1a, Vegfa, Jun, and Stat3 in BZD group were significantly reduced, while the levels of Casp3 and Trp53 were significantly increased. There was no significant difference in Esr1 expression between BZD and NC groups. ns: P > 0.05, **P < 0.01, ***P < 0.001.

To test whether BZD can prevent colorectal cancer growth by altering the core genes in the network pharmacology analysis results and the KEGG enrichment pathway-related genes, RT-qPCR was used to measure target protein mRNA expression levels in tumor tissues of NC and BZD group mice. The BZD group had significantly lower Pik3r1, Akt1, Myc, Esr1, Egfr, Hif1a, Vegfa, Jun, and Stat3 mRNA expression levels than the NC group, according to PCR data. Compared to the NC group, the expression levels of Casp3 and Trp53 in the BZD group were significantly increased. There was no difference in the mRNA expression levels of Esr1 between the NC and BZT groups’ tumor tissues (**Figure 13**). Network pharmacology analysis results show that BZD can prevent colorectal cancer progression by acting on critical core genes and KEGG enrichment pathways. Moreover, these core genes (Pik3r1, Akt1, Myc, Egfr, Hif1a, Vegfr, Jun, and Stat3) are closely related to the tumor immune microenvironment, exclusively T cell immune function.

### 3.10 BZD induces T cell activation in tumor-bearing mice

**Figure 14:**
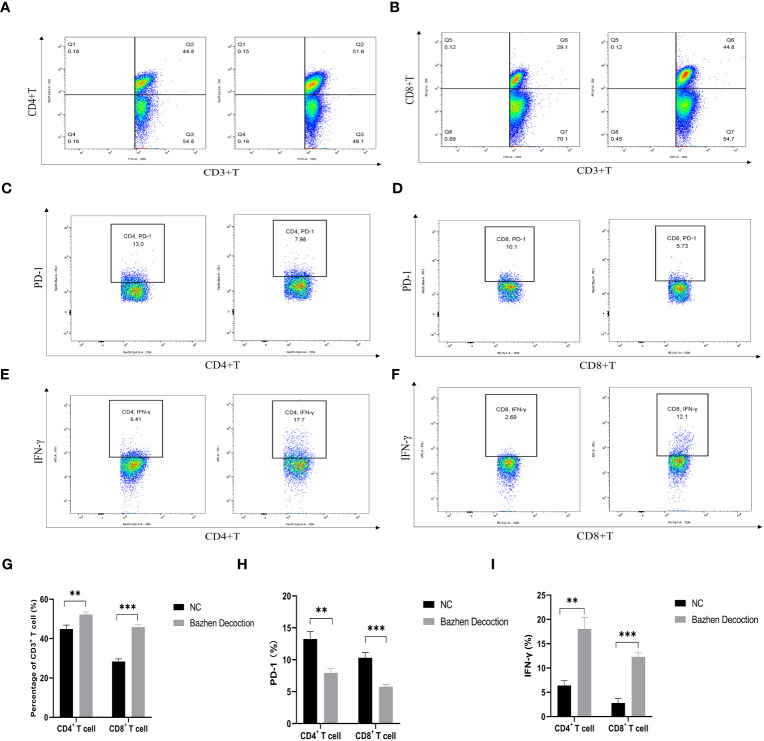
BZD plays an anti-tumor role by significantly increasing the number of tumors infiltrating lymphocytes (TIL) in the body and promoting the activation of T cells. **(A, B, G)** BZD increased the number of CD3^+^CD4^+^ T cells and CD3^+^CD8^+^ T cells in the spleen. **(C, D, H)** BZD reduced the number of CD4^+^PD-1^+^ T cells and CD8^+^PD-1^+^ T cells in the spleen. **(E, F, I)** BZD facilitates the activation of CD4+ and CD8+ T cells within the spleen and increases the levels of IFN-γ within the body. **P < 0.01, ***P < 0.001.

BZD, a traditional formula in the “Fuzheng Guben” treatment principle, can supplement qi, nourish the blood, and enhance the immune system. To clarify the mechanism of the BZD anti-tumor effect, we conducted a flow cytometric analysis of spleen tissues from two groups of mice. The results showed that compared to the control group, BZD increased the proportion of CD3^+^CD4^+^ and CD3^+^CD8^+^ T cells in the spleen (**Figures 14A, B, G**) and decreased the proportion of exhausted T cells (CD4^+^PD-1^+^ and CD8^+^PD-1^+^T cells) in the spleen (**Figures 14C, D, H**). Furthermore, BZD promoted the activation of CD4^+^ and CD8^+^ T cells in the spleen and increased the level of IFN-γ in the body (**Figures 14E, F, I**). These data imply that BZD can reduce the number of exhausted T cells in the spleen of tumor-bearing mice and upregulate IFN-γ to elicit an anti-tumor immune response by infiltrating and activating effector T cells.

### 3.11 BZD promotes T-cell infiltration in tumor tissue of tumor-bearing mice

**Figure 15:**
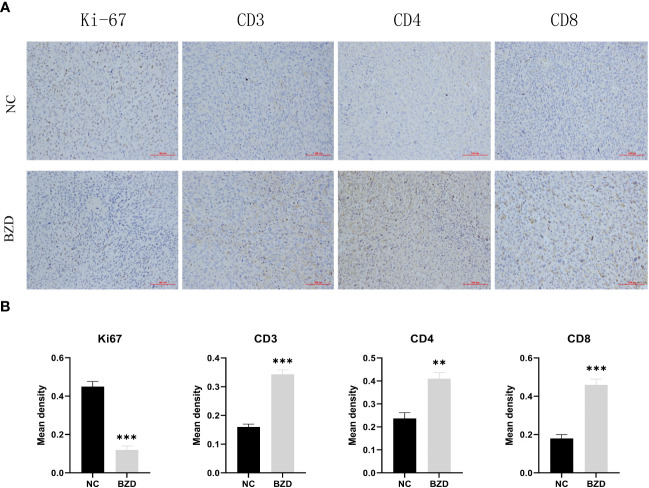
BZD significantly increased the number of Tumor-infiltrating lymphocytes (TIL) *in vivo* and inhibited the expression of Ki67. **(A)** An immunohistochemistry assay is used to detect the CD3, CD4, CD8, and Ki67 expression levels in transplanted tumors (×200). **(B)** Histogram showing the expression levels of CD3, CD4, CD8, and Ki67 in tumor tissues of the control group and BZD group. **P < 0.01, ***P < 0.001.

To further explore the BZD effects on the tumor microenvironment, we performed immunohistochemical staining on the tumor tissues of two groups of mice. In mice’s tumor tissues, the BZD group had more CD3^+^, CD4^+^, and CD8^+^ T cells than the control group. Moreover, the Ki67 staining in the control group was enhanced, indicating that BZD inhibited tumor cell proliferation (**Figure 15**). The results indicate that BZD can promote T cell infiltration in tumor tissues, enhance anti-tumor immune response, and suppress colorectal cancer progression.

## 4 Discussion

CRC accounts for 10% of all cancer cases, ranking third in men and second in women (49, 50). In 2020, there were an estimated 1.9 million new cases and 900,000 deaths globally, rendering it the second leading cause of cancer-related deaths. CRC incidence, mortality, and healthcare services are global public health issues (51). At the time of diagnosis, approximately 20% of CRC patients have already experienced metastasis, and 50% of early-stage patients will eventually develop metastasis (52). Locally advanced rectal and metastatic colorectal cancer (CRC) patients have a poor long-term prognosis, and CRC treatment remains difficult (53). Current treatment modalities involve a multimodal approach, including surgery, radiotherapy, and chemotherapy. However, despite these interventions, recurrence, and metastasis rates remain high. Traditional Chinese medicine has a long history of effectiveness in treating complicated ailments like severe infectious diseases, cardiovascular diseases, and malignant tumors. Traditional Chinese medicine’s characteristics encompass many ingredients, targets, and synergistic effects. Inducing cell apoptosis and autophagy, inhibiting tumor cell proliferation, suppressing epithelial-mesenchymal transition and angiogenesis, modulating chemotherapy resistance, tumor metabolism, and tumor immune regulation may treat colorectal cancer (54–56). In contrast to the adverse effects associated with chemotherapy drugs, including bone marrow suppression, reduced blood cell count, gastrointestinal reactions, liver function impairment, and alopecia, the use of traditional Chinese medicine for treating colorectal cancer exhibits a lower incidence of side effects and drug resistance (57, 58).

BZD, a classic formula utilized in the treatment principle of “Fuzheng Guben,” has been observed to possess the ability to nourish qi and blood. Clinical studies have demonstrated its efficacy in treating various solid tumors. BZD’s mechanism in CRC treatment is unknown. This study is the first to employ network pharmacology and molecular docking to anticipate the core components, core targets, and likely mechanisms of BZD in colorectal cancer treatment. Subsequently, the *in vitro* anticancer effect of BZD was validated using two human colorectal cancer cell lines (HCT116 and SW620). Subsequently, we proceeded to validate the BZD efficacy in inhibiting the progression of colorectal cancer *in vivo* using the MC38 subcutaneous tumor mouse model. We used qRT-PCR to assess gene expression changes in tumor tissues from the control and BZD groups to understand the processes. Additionally, we conducted flow cytometry analysis of the spleen and immunohistochemistry of the tumor tissue to investigate further the relevant mechanism of BZD in treating colorectal cancer. Finally, we evaluated the BZD organ toxicity through HE staining.

In this study, we screened each herb in BZD through ADME to obtain active drug components. Additionally, 258 drug targets were retrieved from databases. We obtained 761 targets correlated to colorectal cancer from the TTD, Drugbank, GeneCards, and DisGeNET databases. By intersecting the targets of drug components with those of colorectal cancer, we obtained 98 overlapping targets. In the herb-ingredient-target network, quercetin, kaempferol, licochalcone A, naringenin, isorhamnetin, 3 ‘- Hydroxy-4’ - O-Methylgabridin, Myricanone, isoliquiritigenin, formonetin, and Glepidotin A have more therapeutic targets for colorectal cancer than other ingredients; thus, they may be the core effector components of BZD in treating colorectal cancer. Quercetin, kaempferol, licochalcone A, naringenin, isorhamnetin, myricanone, and isoliquiritigenin are flavonoid compounds, while formononetin belongs to the isoflavone class. To fight cancer, Quercetin regulates several biological processes, including cell death, autophagy, angiogenesis, metastasis, cell cycle, proliferation, and anti-tumor immune activation (59–64). Quercetin weakens the inhibitory effect of PD-L1 on T cells by inhibiting the PD-1/PD-L1 interaction, promoting the CD8, GZMB, and IFN-γ expression in mouse tumor tissues, and enhancing the anti-tumor immune response (65). Kaempferol is a major flavonoid glycoside with multiple anti-cancer mechanisms (66). It can cause G2/M phase arrest and block cell death in colorectal cancer cells, preventing their proliferation (67). Furthermore, kaempferol can enhance the anti-tumor immune response, synergistically creating a favorable tumor immune microenvironment with radiotherapy and chemotherapy (68). Kaempferol can also enhance anti-tumor immune function by targeting multiple immune-related molecules (69). Kaempferol treatment effectively prevents the decrease of CD4^+^ T cells and CD8^+^ T cells in mouse blood induced by cold stress (70). Kaempferol increases NKT and CD8^+^ T cells and decreases MDSC cells, preventing mouse tumor growth (71). Furthermore, kaempferol is vital in overcoming 5-Fu resistance by inhibiting the glycolysis process in resistant colorectal cancer cells (72). Licochalcone A can inhibit the colorectal cancer cell line HCT116 proliferation by promoting G0/G1 phase arrest, cell apoptosis, and high ROS production (73). Licochalcone A reduces DNA synthesis dose-dependently, thereby inhibiting the mouse colon cancer cells’ CT-26 proliferation and alleviating liver and kidney function damage caused by cisplatin treatment (74). LCA increases T cell anti-tumor efficacy by decreasing PD-L1 expression (75). Licochalcone A therapy can boost cytotoxic T lymphocyte activity and kill tumor cells (76). Other studies have shown that licochalcone A promotes T and B cell proliferation in mice’s spleen and whole blood by activating the IL-17 signaling pathway and improving cognitive ability (77). Naringenin can inhibit cancer progression through various mechanisms, like inducing apoptosis, cell cycle arrest, inhibiting angiogenesis, and modifying various signaling pathways, including Wnt/β- Catenin, PI3K/Akt, NF- κB and TGF- β Pathway (78). Naringenin can enhance CD169 macrophages in lymph nodes of mice with oral squamous cell carcinoma (OSCC) and suppress tumor growth through T-cell-mediated anti-tumor immune activity (79). Moreover, in the mouse colon adenocarcinoma model, Naringenin induced more CD103 DC to infiltrate the tumor, promoted the CD8^+^ T cell activation, and enhanced the performance of the E7 vaccine against TC-1 mouse cancer treatment (80). After breast cancer surgery, naringenin therapy reduces lung metastases and extends mouse life. Flow cytometry demonstrated that Naringenin-treated mice had less regulatory T cells, activated CD8^+^ and CD4^+^ T cells, and IFN-γ. The secretion level of IL-2 has significantly increased (81). Isorhamnetin can block the cell cycle, inhibit proliferation and induce apoptosis by down-regulation of the Bcl-2 gene, upregulation of the Bax gene, inhibition of telomere activity, and reduction of related protein expression (82). Myristone induces apoptosis in two types of cancer cells (HeLa and PC3) by activating caspase and downregulating NF-KB and STAT3 signaling cascade inhibits tumor cell proliferation (83). Myristone has a significant dose-dependent sexual inhibition effect on human lung cancer cell A549 and can promote the apoptosis of lung cancer cells (84). Myristone causes HepG2 apoptosis through ROS generation, mitochondrial membrane depolarization, early cytochrome c release, HSP70 downregulation, and caspase cascade activation (85). Isoliquiritigenin is a natural pigment with a simple Chalcone structure, which can be separated from the root of licorice and is considered a potential Natural product. It is reported that isoliquiritigenin has therapeutic potential for many cancer cell lines, including leukemia, gastrointestinal, breast, colon, ovarian, lung, and melanoma (86). Isoliquiritigenin mediates HIF-1 α stability and inhibits the glycolysis of colorectal cancer cells, thereby inhibiting the proliferation of colon cancer cells (87). Isoliquiritigenin affects the metabolic pathways of tumor cells and inhibits colorectal cancer growth by triggering cell cycle arrest, apoptosis, and autophagy and altering tumor cell metabolism (86, 88). Formononetin is a common component in legumes, particularly rich in *Trifolium pratense L*. and *Astragalus membranaceus*. Formononetin can inhibit tumor cell proliferation by inducing cell cycle arrest and induce cell apoptosis by regulating Bax, Bcl-2, and caspase-3 proteins. Moreover, formononetin inhibits cell invasion by regulating Vascular endothelial growth factor (VEGF) and Fibroblast growth factor 2 (FGF2). Anthocyanin’s anticancer properties can be enhanced by synergistic effects with other chemotherapy drugs (89). Other studies have shown that formononetin enhances T cells’ activity and killing ability by inhibiting the PD-L1 expression on the cell surface, thereby inhibiting tumor proliferation, angiogenesis, migration, and invasion (90). However, there are no reports on treating cancer with the two active ingredients, 3 ‘- Hydroxy-4’ - O-Methylgabridin and Glepidotin A, which need to be explored in future research. Besides inhibiting the proliferation and invasion of colorectal cancer cells and promoting colorectal cancer cells apoptosis, the five core effector components (quercetin, kaempferol, licochalcone A, naringenin, and formaronetin) of BZD can also enhance the immune function against colorectal cancer by promoting the T cells activation and killing ability.

In the PPI network, we identified 10 key genes, namely AKT1, MYC, CASP3, ESR1, EGFR, HIF-1A, VEGFA, JUN, INS, and STAT3, which may be the core targets of BZD therapy for CRC. AKT1, a proto-oncogene in the serine/threonine kinase family, regulates tumor cell proliferation, survival, and metabolism through inflammation and metabolism-related signaling pathways (91). About 70% of colorectal cancers exhibit highly activated AKT, closely associated with cancer development. Abu-Eid found that after using AKT inhibitors, tregs were more susceptible to inhibition, increasing the number of CD8^+^ T cells in tumor tissue and improving control of tumor lesions (92). Modified-Bu-zhong-yi-qi decoction (mBYD) directly enhances T lymphocyte proliferation and activation by blocking the PI3K/AKT signaling pathway and suppressing cancer cell PD-L1 expression, ultimately preventing gastric cancer growth (93). The MYC oncogene is part of the gene superfamily, and its product is commonly activated in human cancers (94, 95). The mechanisms by which MYC activation promotes tumor progression mainly involve cell proliferation, cell invasion, metabolic reprogramming, genomic instability, angiogenesis, and immune evasion (96, 97). MYC can reshape the tumor microenvironment, evading host immune responses (98). Myc inhibitors decrease the stable state of regulatory T (Treg) cells in tumors and the differentiation of resting treg (rTreg) to activated Treg (aTreg), which activates CD8^+^T cells and induces anti-tumor immune response (99). Cysteine Aspartic protease 3 (CASP3) serves as the primary mediator of apoptosis in tumor cells when exposed to cytotoxic drugs, radiotherapy, or immunotherapy, making it a commonly employed marker for assessing the efficacy of cancer treatment. Targeting CASP3 therapeutically increases tumor cell sensitivity to chemotherapy and radiotherapy and inhibits cancer cell invasion and metastasis (100). Studies have shown that ESR1 mutations can promote tumor progression and metastasis. During treating metastatic estrogen receptor (ER) positive breast cancer with Aromatase inhibitors, ESR1 mutations are a common mechanism of hormone therapy resistance (101). Epidermal growth factor receptor (EGFR) is key for cell proliferation, differentiation, and survival (102). EGFR is overexpressed in 25–77% of colorectal cancer, which is related to the poor prognosis of cancer patients (103–105). Besides directly promoting tumor cell proliferation, EGFR can also serve as a modulator for tumor immune monitoring, promoting the PD-L1 expression by activating the JAK/STAT3 signaling pathway, inducing T cell apoptosis and immune escape. EGFR Tyrosine kinase inhibitors (TKIs) can enhance the effect of MHC class I and II antigens on IFN-γ to increase CD8^+^T cells and DC cells levels, eliminate FOXP3^+^Tregs, inhibit Macrophage polarization to M2 phenotype, and reduce the PD-L1 expression in cancer cells (106).

HIF-1α is a transcription activator that is reliant on oxygen levels. Through various mechanisms, including angiogenesis, cellular proliferation and survival, metabolic reprogramming, invasion and metastasis, maintenance of cancer stem cells, induction of genetic instability, and resistance to therapeutic interventions, the upregulation of its downstream genes contributes to the growth of tumors. Consequently, modulating the downstream signaling molecule of HIF-1α presents an opportunity to regulate the initiation and progression of tumors (107). Hypoxia increases the PD-L1 expression and induces apoptosis of cytotoxic T lymphocytes (CTLs), thereby promoting the immune escape of tumor cells (108, 109). According to research findings, the inhibitor echinocandin of HIF-1 α has been observed to augment the immune tolerance function of the PD-1/PD-L1 checkpoint in normal tissues. Additionally, it has been found to enhance both the quantity and efficacy of Tumor-infiltrating lymphocytes, thereby leading to a safer and more efficacious approach to immunotherapy (110). VEGF-A is a highly conserved secretory signaling protein known for its role in vascular development and angiogenesis (111). Targeting VEGF is a feasible strategy for preventing tumor growth and metastasis (112). Research has indicated that VEGF-A is critical in triggering tumor immunosuppression and boosting angiogenesis. VEGF-A encourages immune-suppressive cell growth, prevents T-cell infiltration of malignancies, and encourages T-cell depletion (113). In a mouse model of colorectal cancer (CT26) targeting VEGFR therapy, it was found that targeting VEGF-a-VEGFR can reduce the co-expression of inhibitory receptors (PD-1, Tim-3, CTLA-4, and Lag-3) related to T cell failure and restore the CD8^+^ T cells infiltration into the tumor to produce IFN- γ ability (114). In mice, Sunitinib increased the CD4^+^ and CD8^+^ T cells proportion in Tumor-infiltrating lymphocytes (115). C-Jun is the most widely studied protein in the activator protein-1 (AP-1) complex and participates in many cell activities, like proliferation, apoptosis, survival, tumorigenesis, and tissue Morphogenesis (116). As an early stage of human colorectal cancer development, adenomas and adenocarcinomas have significantly higher levels of c-Jun protein expression (117). JNK1 is highly expressed in HCT116 colon and PANC1 pancreatic cancer cells. Using licorice chalcone A or knocking down JNK1 expression can inhibit the proliferation and colony formation of colon cancer and pancreatic cancer cells (118). STAT3 is a tumor-promoting oncogene shown in several tumors and is intimately linked to inflammation and immunity (119). In the adaptive immune subgroup, elevated STAT3 activity can inhibit the aggregation of effector T cells, thereby inhibiting their anti-tumor effects (120–122). Targeting STAT3 may reduce tumor cell-intrinsic proliferation, increase tumor-infiltrating immune cell anti-tumor activity, and improve TME immune suppression (123). The molecular docking results indicate that the central genes and core components possess significant binding potential, indicating that BZD could inhibit colorectal cancer progression by targeting these key genes.

GO enrichment analysis shows that BZD treats colorectal cancer by regulating biological processes like cell proliferation, apoptosis, energy metabolism, and immune response. KEGG enrichment analysis results showed that PI3K-AKT, T-cell receptor, P53, and VEGF signaling pathway may be the potential key pathway of BZD in treating colorectal cancer. Numerous cancer forms hyperactivate or modify the PI3K-AKT signaling system, which controls cellular activities like survival, proliferation, growth, metabolism, angiogenesis, metastasis, and immune response (124, 125). PI3K activity suppression can decrease PD-L1 expression and heightened IFN-γ mediated anti-tumor effects (126). A specific inhibitor (IPI-549) that targets PI3K-γ has the potential to remodel the immune milieu within the tumor microenvironment (TME) and facilitate CTL-mediated tumor regression (127). The T-cell receptor (TCR) signaling pathway is vital in promoting the development, homeostasis, proliferation, differentiation of T cells, and the production of cytokines, thereby eliciting a robust anti-tumor immune response (128). The tumor cell cycle, aging, apoptosis, metabolism, and immune response are regulated by P53, a well-studied tumor suppressor gene (129, 130). Multiple plant components can inhibit the progression of colorectal cancer cells by upregulating p53 to induce G2/M phase arrest and cell apoptosis (131–133). The VEGF-VEGFR signaling pathway is widely recognized as the most crucial pathway for inducing angiogenesis. Inhibition of this cascade reaction has proven to be effective in the treatment of tumors (134). Extensive research has demonstrated that targeting the VEGF signaling pathway increases tumor CD8^+^T cell invasion and activation, boosting T cell cytokine output (135–137).

To explore the anticancer mechanism of BZD, we first compared the expression differences of key genes (core genes in PPI and related genes in KEGG enrichment pathway in network pharmacology analysis) in mouse tumor tissues between the control and BZD group through qRT-PCR experiments. The experimental results showed that compared with the control group, the expression of Pik3r1, Akt1, Myc, Egfr, Hif1a, Vegfr, Jun, and Stat3 genes in the tumor tissue of mice in the BZD group was significantly reduced, and the Casp3 and Trp53 genes expression was significantly increased; this is consistent with the predicted results of network pharmacology. The results show that BZD can suppress colorectal cancer growth by modulating the above essential genes. Moreover, these key genes are closely related to the tumor immune microenvironment, uniquely T cell immune function. As a classic formula utilized in the “Fuzheng Guben” treatment principle, the main effect of BZD is to supplement both qi and blood and enhance the body’s immune system. Considering BZD’s main efficacy, qRT-PCR, and the T-cell receptor signal pathway in KEGG enrichment analysis, we used flow cytometry to detect T lymphocyte subsets in tumor-bearing mice’s spleens. The results showed that compared to the control group, BZD significantly increased the number of CD4^+^ and CD8^+^T cells in the spleen of tumor-bearing mice, promoted T cell activation, and increased IFN-γ level. Furthermore, BZD reduced the number of PD-1^+^CD4^+^ and PD-1^+^CD8^+^ T cells. IFN-γ is the main cytokine that enhances the host’s anti-tumor immune function and is stably produced during CD8^+^ T cell differentiation into cytotoxic T lymphocytes and memory cells in response to TCR stimulation. Therefore, IFN-γ elevated expression effectively indicates CD8^+^T cell activation and anti-tumor immune response. The lower PD-1^+^CD4^+^T cells and PD-1^+^CD8^+^T cell populations showed a decrease in depleted T cell proportion. We performed immunohistochemistry labeling on the tumor tissues of two groups of mice to determine T-cell infiltration and cancer cell growth. The results showed that compared to the control group, the infiltration of CD3^+^, CD4^+^, and CD8^+^ T cells in the tumor tissue of the BZD group increased while the Ki67 expression decreased. Therefore, BZD can enhance the anti-colorectal cancer immune function by increasing T cell infiltration in tumor tissue. These immunological results are not only consistent with the tumor volume and weight in mice, but also consistent with the potential mechanisms of tumor inhibition by previous “Fu Zheng Gu Ben” formula, Liu Jun Zi Decoction, and Bu Shen Hui Yang formula (138, 139). Additionally, pathological results showed that BZD does not cause any organ damage and is considered safe.

To our knowledge, this study is the first to explore the efficacy and mechanism of BZD in the treatment of CRC. In this study, we accomplished the following work: (1) The core components, key targets, and signaling pathways of BZD in the treatment of CRC were analyzed using network pharmacology. (2) This study validated the good binding ability of core drug components to key targets through molecular docking. (3) This study verified through *in vitro* experiments that BZD inhibits the proliferation and invasion of CRC cells in a time- and dose-dependent manner, while promoting apoptosis of CRC cells. (4) This study verified through animal experiments that BZD can significantly inhibit the progression of CRC and has no organ toxicity. (5) Our experiments demonstrated that BZD inhibit CRC by regulating the core targets and signaling pathways in the tumor microenvironment. (6) BZD promotes T cell infiltration and activation in the spleen and tumor tissue of tumor bearing mice, inhibits PD-1 expression on the surface of T cells, restores T cell killing ability, and enhances immune function against colorectal cancer. However, it is undeniable that there are still deficiencies in our experiment. Although we have confirmed the anticancer effect of BZD on CRC, its underlying substance basis is not yet clear. Furthermore, the mechanism by which BZD inhibits CRC is not thorough enough. Therefore, in future studies, we will further explore the main active components and more detailed mechanisms of BZD in inhibiting CRC.

## 5 Conclusion

BZD treats CRC through multiple components, targets, and metabolic pathways. Our research confirmed BZD’s anti-colorectal cancer efficacy for the first time and studied its essential components, core targets, and potential mechanism using network pharmacology, molecular docking, and experiments. BZD can reverse the abnormal expression of PI3K, AKT, MYC, EGFR, HIF-1A, VEGFR, JUN, STAT3, CASP3, and TP53 genes in the tumor microenvironment and inhibit the progression of colorectal cancer by regulating the signaling pathways such as PI3K-AKT, P53, and VEGF. In addition, BZD can promote infiltration and activation of T cells in tumor-bearing mice, suppress the expression of PD-1 on T cell surface, restore T cell cytotoxicity, enhance anti-tumor immune response, and inhibit the progression of colorectal cancer. Among them, quercetin, kaempferol, licochalcone A, naringenin, and formaronetin are more highly predictive components related to the T cell activation in colorectal cancer mice. This study may offer new perspectives to the treatment of colorectal cancer and provide new ideas for exploring new anticancer drugs. However, the main active ingredients and more detailed mechanisms of BZD inhibition on CRC still need to be further explored in future studies.

## Data availability statement

The original contributions presented in the study are included in the article/supplementary materials, further inquiries can be directed to the corresponding author/s.

## Ethics statement

The Ethics Committee of Beijing Shijitan Hospital Affiliated to Capital Medical University reviewed and granted approval for this study (The ethical approval permit numbers are SJTKY11-1X-2021(59)). The study was conducted in accordance with the local legislation and institutional requirements.

## Author contributions

SL and BR conceived and designed this study. SL, XS, ZZ, and QL conducted network pharmacology analysis. HT, BW, and JQ conducted molecular docking. JY, FS, and YT conducted statistical analysis. ZD and CL completed the figures. SL and RX completed *in vitro* and *in vivo* experiments. SL, XS, and ZZ wrote the manuscript. BR, PY, and ZY participated in the review and revision of the manuscript. All authors contributed to the article and approved the submitted version.

## References

[1] Sung H Ferlay J Siegel RL Laversanne M Soerjomataram I Jemal A. Global cancer statistics 2020: GLOBOCAN estimates of incidence and mortality worldwide for 36 cancers in 185 countries. CA Cancer J Clin (2021) 71(3):209–49. doi: 10.3322/caac.21660 33538338

[2] Kuipers EJ Grady WM Lieberman D Seufferlein T Sung JJ Boelens PG. Colorectal cancer. Nat Rev Dis Primers (2015) 1:15065. doi: 10.1038/nrdp.2015.65 27189416 PMC4874655

[3] Brown KGM Solomon MJ. Progress and future direction in the management of advanced colorectal cancer. Br J Surg (2018) 105(6):615–7. doi: 10.1002/bjs.10759 29652083

[4] Matsuda T Yamashita K Hasegawa H Oshikiri T Hosono M Higashino N. Recent updates in the surgical treatment of colorectal cancer. Ann Gastroenterol Surg (2018) 2(2):129–36. doi: 10.1002/ags3.12061 PMC5881369 29863145

[5] Ooft SN Weeber F Dijkstra KK McLean CM Kaing S van Werkhoven E. Patient-derived organoids can predict response to chemotherapy in metastatic colorectal cancer patients. Sci Trans Med (2019) 11(513):eaay2574. doi: 10.1126/scitranslmed.aay2574 31597751

[6] Halama N Zoernig I Berthel A Kahlert C Klupp F Suarez-Carmona M. Tumoral immune cell exploitation in colorectal cancer metastases can Be targeted effectively by anti-CCR5 therapy in cancer patients. Cancer Cell (2016) 29(4):587–601. doi: 10.1016/j.ccell.2016.03.005 27070705

[7] Dekker E Rex DK. Advances in CRC prevention: screening and surveillance. Gastroenterology (2018) 154(7):1970–84. doi: 10.1053/j.gastro.2018.01.069 29454795

[8] Xu HY Zhang YQ Liu ZM Chen T Lv CY Tang SH. Etcm: An encyclopaedia of traditional Chinese medicine. Nucleic Acids Res (2019) 47(D1):D976–82. doi: 10.1093/nar/gky987 PMC6323948 30365030

[9] Luo X-Y Wu K-M He X-X. Advances in drug development for hepatocellular carcinoma: clinical trials and potential therapeutic targets. J Exp Clin Cancer research: CR (2021) 40(1):172. doi: 10.1186/s13046-021-01968-w 34006331 PMC8130401

[10] Kong M-Y Li L-Y Lou Y-M Chi H-Y Wu J-J. Chinese herbal medicines for prevention and treatment of colorectal cancer: From molecular mechanisms to potential clinical applications. J Integr Med (2020) 18(5):369–84. doi: 10.1016/j.joim.2020.07.005 32758397

[11] Song YJ Bao JM Zhou LY Li G Sng KS Wang YJ. An analysis of the antineuropathic effects of qi she pill based on network pharmacology. Evidence-Based Complement Altern Med (2020) 2020:1–15. doi: 10.1155/2020/7193832 PMC7222608 32454869

[12] Song Y Wang H Pan Y Liu T. Investigating the multi-target pharmacological mechanism of Hedyotis diffusa willd acting on prostate cancer: a network pharmacology approach. Biomolecules (2019) 9(10):591. doi: 10.3390/biom9100591 31600936 PMC6843553

[13] Ren B Tan L Xiong Y Ji W Mu J Pei Y. Integrated analysis of the mechanisms of da-chai-hu decoction in type 2 diabetes mellitus by a network pharmacology approach. Evidence-Based Complement Altern Med (2020) 2020(10027):1–21. doi: 10.1155/2020/9768414 PMC7204321 32419835

[14] Meng Z Liu X Wu J Zhou W Wang K Jing Z. Mechanisms of compound kushen injection for the treatment of lung cancer based on network pharmacology. Evidence-Based Complement Altern Med (2019) 2019:1–15. doi: 10.1155/2019/4637839 PMC6558614 31275410

[15] Meng XY Zhang HX Mezei M Cui M. Molecular docking: a powerful approach for structure-based drug discovery. Curr Comput Aided Drug Des (2011) 7(2):146–57. doi: 10.2174/157340911795677602 PMC3151162 21534921

[16] Guo W Huang J Wang N Tan HY Cheung F Chen F. Integrating network pharmacology and pharmacological evaluation for deciphering the action mechanism of herbal formula zuojin pill in suppressing hepatocellular carcinoma. Front Pharmacol (2019) 10:1185. doi: 10.3389/fphar.2019.01185 31649545 PMC6795061

[17] Liu J Liu J Tong X Peng W Wei S Sun T. Network pharmacology prediction and molecular docking-based strategy to discover the potential pharmacological mechanism of huai hua san against ulcerative colitis. Drug Des Devel Ther (2021) 15:3255–76. doi: 10.2147/DDDT.S319786 PMC8326529 34349502

[18] Lahans T. Integrating Chinese and conventional medicine in colorectal cancer 13 treatment. Integr Cancer Therapies (2007) 6(1):89. doi: 10.1177/1534735406298991 17351031

[19] Cao X Yan X. Clinical efficacy of Bazhen Tang combined with radiotherapy and chemotherapy in the treatment of cervical cancer and its impact on patient immune function, cancer-related fatigue, and toxic side effects. Clin Med Res Pract (2022) 7(12):138–41. doi: 10.19347/j.cnki.2096-1413.20221203

[20] Yang H Guo J Jing R. The effect of modified Bazhen Tang combined with moxibustion on tolerance, analgesic effect, and quality of life in patients with advanced cervical cancer undergoing radiotherapy and chemotherapy. Chin J Exp Prescript (2018) 24(9):173–8.

[21] Xu AH Gong YX Gu WR Wang XW. Comparison of the effect of sijunzi decoction, siwu decoction and bazhen decoction on immune function in mice. Zhongguo Zhong yao za zhi = Zhongguo zhongyao zazhi = China J Chin mater Med (1993) 18(4):240–2, 256. 8216794

[22] Liu X Wang X Li Z Chen,Y Chen J. *In vitro* study for detecting the effects of Bazhen decoction on proliferation and activation of T lymphocytes. Sheng wu yi xue gong cheng xue za zhi = J Biomed Eng = Shengwu yixue gongchengxue zazhi (2010) 27(4):855–8. 20842859

[23] Wang H-x Li J-p. Effects of modified bazhen decoction in assistant with enteral nutrition on the growth hormone, the nutritional state, and the immune function in patients with gastric cancer after operation. Zhongguo Zhong xi yi jie he za zhi Zhongguo Zhongxiyi jiehe zazhi = Chin J integrated traditional Western Med (2011) 31(10):1317–21. 22097196

[24] Shi T Chen B Liu C Lu K. Study on huangqi bazhen decoction on relieving chemotherapy intestinal mucositis in capecitabine gavage mice. Contrast media Mol Imaging (2022) 2022:3826080. doi: 10.1155/2022/3826080 36247864 PMC9534644

[25] Tian Y Xiang Y Wan G Wan D Zhu H. Effects and mechanisms of Bazhen decoction, Siwu decoction, and Sijunzi decoction on 5-fluorouracil-induced anemia in mice. J traditional Chin Med = Chung i tsa chih ying wen pan (2016) 36(4):486–95. 10.1016/s0254-6272(16)30066-8 28459513

[26] Lu X Zheng Y Wen F Huang W Shu P. Effectiveness and Safety of Oral Chinese Patent Medicines Combined with Chemotherapy for Gastric Cancer: A Bayesian Network Meta-Analysis. Evidence-based complementary and alternative medicine. eCAM (2020) 2020:8016531. doi: 10.1155/2020/8016531 32908569 PMC7471790

[27] Niu ZE Jing DX Xu CY. Clinical effect of bazhen decoction combined with sequential treatment of chemotherapy on acute lymphoblastic leukemia patients with deficiency of qi and yin. Zhongguo Shi Yan Xue Ye Xue Za Zhi (2022) 30(1):119–25. doi: 10.19746/J.CNKI.ISSN.1009-2137.2022.01.19 35123613

[28] Zhang J Xu J Jin Z. The effect of modified Bazhen decoction combined with chemotherapy on the nutritional status and immune function of postoperative colon cancer patients. J Anhui Univ Traditional Chin Med (2023) 42(03):9–13.

[29] Jiao J Qu X Ding B. The therapeutic effect of modified Bazhen Tang combined with chemotherapy on postoperative patients with gastric cancer. J Pract Clin Med (2019) 23(15):86–9.

[30] Xu N Xiao M Luo T Ma H Liu Y. Clinical efficacy of Bazhen Tang as an adjuvant chemotherapy in the treatment of advanced non-small cell lung cancer in the elderly. Clin Med Res Pract (2021) 6(19):128–129+139. doi: 10.19347/j.cnki.2096-1413.202119040

[31] Chen B Sun J Wang H. Bazhen Decoction combined with neoadjuvant chemotherapy in the treatment of 70 cases of breast cancer. Natl Med Forum (2021) 36(02):37–8. doi: 10.13913/j.cnki.41-1110/r.2021.02.016

[32] Wu Y Chen L. Modified Bazhen Tang combined with radiotherapy for the treatment of advanced cervical cancer and its effect on the cellular immune level and nutritional status of patients. Shaanxi Traditional Chin Med (2022) 43(08):1052–5.

[33] Xu Z Jin Y Tao Z Yu X Fang M Tong X. Clinical observation of modified Bazhen Tang combined with capecitabine in the maintenance treatment of elderly patients with advanced colon cancer. Chin J Traditional Chin Med Sci Technol (2022) 29(06):1123–5.

[34] Zhou Y Lv X. Observation on the efficacy of Bazhen Tang combined with chemotherapy in the treatment of advanced colon cancer. World J Integrated Traditional Chin Western Med (2020) 15(08):1524–7. doi: 10.13935/j.cnki.sjzx.200833

[35] Xu Z Wang C Luan Z Zhang D Dong B. Exploring the potential targets of the *Abrus cantoniensis* Hance in the treatment of hepatitis E based on network pharmacology. Front Vet Sci (2023) 10:1155677. doi: 10.3389/fvets.2023.1155677 37035802 PMC10076809

[36] Wishart DS Feunang YD Guo AC Lo EJ Marcu A Grant JR. DrugBank 5.0:a major update to the DrugBank database for 2018. Nucleic Acids Res (2018) 46(D1):D1074–82. doi: 10.1093/nar/gkx1037 PMC5753335 29126136

[37] Mok SRS Mohan S Grewal N Elfant AB Judge TA. A genetic database can be utilized to identify potential biomarkers for biphenotypic hepatocellular carcinoma-cholangiocarcinoma. J Gastrointest Oncol (2016) 7(4):570–9. doi: 10.21037/jgo.2016.04.01 PMC4963376 27563447

[38] Li YH Yu CY Li XX Zhang P Tang J Yang Q. Therapeutic target databaseupdate 2018: enriched resource for facilitating bench-to-clinicresearch of targeted therapeutics. Nucleic Acids Res (2018) 46(D1):D1121–7. doi: 10.1093/nar/gkx1076 PMC5753365 29140520

[39] Piñero J Queralt-Rosinach N Bravo A Deu-Pons J Bauer-Mehren A Baron M. DisGeNET: a discovery platform for the dynamical exploration of human diseases and their genes. Database (2015) 2015(0):bav028. doi: 10.1093/database/bav028 25877637 PMC4397996

[40] Franz M Lopes CT Huck G Dong Y Sumer O Bader GD. Cytoscape.js: a graph theory library for visualisation and analysis. Bioinformatics (2016) 32(2):309–11. doi: 10.1093/bioinformatics/btv557 PMC4708103 26415722

[41] Wang F Yuan C Wu HZ Liu B Yang YF. Bioinformatics, molecular docking and experiments in vitro analyze the prognostic value of CXC chemokines in breast cancer. Front Oncol (2021) 11:665080. doi: 10.3389/fonc.2021.665080 34123826 PMC8189319

[42] Chen Y Zhou Q Zhang H Xu L Lu L Shu B. Qingdai Decoction suppresses prostate cancer growth in lethal-stage prostate cancer models. J Ethnopharmacol (2023) 308:116333. doi: 10.1016/j.jep.2023.116333 36863640

[43] Zhang M Wu W Huang C Cai T Zhao N Liu S. Shuxie-1 decoction alleviated CUMS -induced liver injury via IL-6/JAK2/STAT3 signaling. Front Pharmacol (2022) 13:848355. doi: 10.3389/fphar.2022.848355 35462928 PMC9019685

[44] Triplett TA Garrison KC Marshall N Donkor M Blazeck J Lamb C. Reversal of indoleamine 2,3-dioxygenase-mediated cancer immune suppression by systemic kynurenine depletion with a therapeutic enzyme. Nat Biotechnol (2018) 36(8):758–64. doi: 10.1038/nbt.4180 PMC6078800 30010674

[45] Wu Y Hao X Wei H Sun R Chen Y Tian Z. Blockade of T-cell receptor with Ig and ITIM domains elicits potent antitumor immunity in naturally occurring HBV-related HCC in mice. Hepatology (2023) 77(3):965–81. doi: 10.1002/hep.32715 35938354

[46] Dai X Lu L Deng S Meng J Wan C Huang J. USP7 targeting modulates anti-tumor immune response by reprogramming Tumor-associated Macrophages in Lung Cancer. Theranostics (2020) 10(20):9332–47. doi: 10.7150/thno.47137 PMC7415808 32802195

[47] Kan X Zhou G Zhang F Ji H Shin DS Monsky W. Enhanced efficacy of direct immunochemotherapy for hepatic cancer with image-guided intratumoral radiofrequency hyperthermia. J Immunother Cancer (2022) 10(11):e005619. doi: 10.1136/jitc-2022-005619 36450380 PMC9717415

[48] Wu X Zhang H Xing Q Cui J Li J Li Y. PD-1(+) CD8(+) T cells are exhausted in tumours and functional in draining lymph nodes of colorectal cancer patients. Br J Cancer (2014) 111(7):1391–9. doi: 10.1038/bjc.2014.416 PMC4183848 25093496

[49] Siegel RL Miller KD Fuchs HE Jemal A. Cancer statistics, 2022. CA Cancer J Clin (2022) 72:7–33. doi: 10.3322/caac.21708 35020204

[50] Siegel RL Miller KD Goding Sauer A Fedewa SA Butterly LF Anderson JC. Colorectal cancer statistics, 2020. CA Cancer J Clin (2020) 70:145–164. doi: 10.3322/caac.21601 32133645

[51] Global Burden of Disease 2019 Cancer Collaboration Kocarnik JM Compton K Dean FE Fu W Gaw BL Harvey JD. Cancer incidence, mortality, years of life lost, years lived with disability, and disability- adjusted life years for 29 cancer groups from 2010 to 2019: a systematic analysis for the Global Burden of Disease Study 2019. JAMA Oncol (2021) 8(3):420–444. doi: 10.1001/jamaoncol.2021.6987 PMC8719276 34967848

[52] Ciardiello F Ciardiello D Martini G Napolitano S Tabernero J Cervantes A. Clinical management of metastatic colorectal cancer in the era of precision medicine. CA Cancer J Clin (2022) 72(4):372–401. doi: 10.3322/caac.21728 35472088

[53] Shi J Sun Z Gao Z Huang D Hong H Gu J. Radioimmunotherapy in colorectal cancer treatment: present and future. Front Immunol (2023) 14:1105180. doi: 10.3389/fimmu.2023.1105180 37234164 PMC10206275

[54] Lin X Yang X Yang Y Zhang H Huang X. Research progress of traditional Chinese medicine as sensitizer in reversing chemoresistance of colorectal cancer. Front Oncol (2023) 13:1132141. doi: 10.3389/fonc.2023.1132141 36994201 PMC10040588

[55] Chen JF Wu SW Shi ZM Hu B. Traditional Chinese medicine for colorectal cancer treatment: potential targets and mechanisms of action. Chin Med (2023) 18(1):14. doi: 10.1186/s13020-023-00719-7 36782251 PMC9923939

[56] Li W Li C Zheng H Chen G Hua B. Therapeutic targets of Traditional Chinese Medicine for colorectal cancer. J Tradit Chin Med (2016) 36(2):243–9. doi: 10.1016/s0254-6272(16)30034-6 27400481

[57] Fei Z Lijuan Y Xi Y Wei W Jing Z Miao D. Gut microbiome associated with chemotherapy-induced diarrhea from the CapeOX regimen as adjuvant chemotherapy in resected stage III colorectal cancer. Gut Pathog (2019) 11(1):18. doi: 10.1186/s13099-019-0299-4 31168325 PMC6489188

[58] Zimmer P Trebing S Timmers-Trebing U Schenk A Paust R Bloch W. Eight-week, multimodal exercise counteracts a progress of chemotherapy-induced peripheral neuropathy and improves balance and strength in metastasized colorectal cancer patients: a randomized controlled trial. Support Care Cancer (2018) 26(2):615–24. doi: 10.1007/s00520-017-3875-5 28963591

[59] Homayoonfal M Gilasi H Asemi Z Khaksary Mahabady M Asemi R Y ousefi B. Quercetin modulates signal transductions and targets non-coding RNAs against cancer development. Cell Signal (2023) 107:110667. doi: 10.1016/j.cellsig.2023.110667 37023996

[60] Golmohammadi M Elmaghraby DA Ramí rez-Coronel AA Rakhimov N Mohammed SS Romero-Parra RM. A comprehensive view on the quercetin impact on bladder cancer: Focusing on oxidative stress, cellular, and molecular mechanisms. Fundam Clin Pharmacol (2023) 37(5):900–9. doi: 10.1111/fcp.12896 36960597

[61] Lotfi N Y ousefi Z Golabi M Khalilian P Ghezelbash B Montazeri M. The potential anti-cancer effects of quercetin on blood, prostate and lung cancers: An update. Front Immunol (2023) 14:1077531. doi: 10.3389/fimmu.2023.1077531 36926328 PMC10011078

[62] Sethi G Rath P Chauhan A Ranjan A Choudhary R Ramniwas S. Apoptotic mechanisms of quercetin in liver cancer: recent trends and advancements. Pharmaceutics (2023) 15(2):712. doi: 10.3390/pharmaceutics15020712 36840034 PMC9960374

[63] Maugeri A Calderaro A Patanè GT Navarra M Barreca D Cirmi S. Targets involved in the anti-cancer activity of quercetin in breast, colorectal and liver neoplasms. Int J Mol Sci (2023) 24(3):2952. doi: 10.3390/ijms24032952 36769274 PMC9918234

[64] Zhang J Shen L Li X Song W Liu Y Huang L. Nanoformulated codelivery of quercetin and alantolactone promotes an antitumor response through synergistic immunogenic cell death for microsatellite-stable colorectal cancer. ACS Nano (2019) 13(11):12511–24. doi: 10.1021/acsnano.9b02875 31664821

[65] Jing L Lin J Yang Y Tao L Li Y Liu Z. Quercetin inhibiting the PD-1/PD-L1 interaction for immune-enhancing cancer chemopreventive agent. Phytother Res (2021) 35(11):6441–51. doi: 10.1002/ptr.7297 34560814

[66] Imran M Salehi B Sharifi- Rad J Aslam Gondal T Saeed F Imran A. Kaempferol: a key emphasis to its anticancer potential. Molecules (2019) 24(12):2277. doi: 10.3390/molecules24122277 31248102 PMC6631472

[67] Choi JB Kim JH Lee H Pak JN Shim BS Kim SH. Reactive Oxygen Species and p53 Mediated Activation of p38 and Caspases is Critically Involved in Kaempferol Induced Apoptosis in Colorectal Cancer Cells. J Agric Food Chem (2018) 66(38):9960–7. doi: 10.1021/acs.jafc.8b02656 30211553

[68] Huang X Wang Y Yang W Dong J Li L. Regulation of dietary polyphenols on cancer cell pyroptosis and the tumor immune microenvironment. Front Nutr (2022) 9:974896. doi: 10.3389/fnut.2022.974896 36091247 PMC9453822

[69] Hofer S Geisler S Lisandrelli R Nguyen Ngoc H Ganzera M Schennach H. Pharmacological targets of kaempferol within inflammatory pathways-A hint towards the central role of tryptophan metabolism. Antioxid (Basel). (2020) 9(2):180. doi: 10.3390/antiox9020180 PMC7070836 32098277

[70] Jia Z Chen A Wang C He M Xu J Fu H. Amelioration effects of Kaempferol on immune response following chronic intermittent cold-stress. Res Vet Sci (2019) 125:390–6. doi: 10.1016/j.rvsc.2019.08.012 31412308

[71] Qiang D Ci C Liu W Wang J He C Ji B. Inhibitory effect of kaempferol on mouse melanoma cell line B16 *in vivo* and *in vitro* . Adv Dermatol Allergol (2021) 38:498–504. doi: 10.5114/ada.2020.94257 PMC8330872 34377134

[72] Wu H Du J Li C Li H Guo H Li Z. Kaempferol can reverse the 5-fu resistance of colorectal cancer cells by inhibiting PKM2-mediated glycolysis. Int J Mol Sci (2022) 23(7):3544. doi: 10.3390/ijms23073544 35408903 PMC8998549

[73] Wu P Y u T Wu J Chen J. Licochalcone a induces ROS-mediated apoptosis through trxR1 inactivation in colorectal cancer cells. BioMed Res Int (2020) 2020:5875074. doi: 10.1155/2020/5875074 32596335 PMC7275230

[74] Lee CK Son SH Park KK Park JH Lim SS Kim SH. Licochalcone A inhibits the growth of colon carcinoma and attenuates cisplatin-induced toxicity without a loss of chemotherapeutic efficacy in mice. Basic Clin Pharmacol Toxicol (2008) 103(1):48–54. doi: 10.1111/j.1742-7843.2008.00238.x 18484961

[75] Y uan LW Jiang XM Xu YL Huang MY Chen YC Y u WB. Licochalcone A inhibits interferon-gamma-induced programmed death-ligand 1 in lung cancer cells. Phytomedicine (2021) 80:153394. doi: 10.1016/j.phymed.2020.153394 33130472

[76] Liu X Xing Y Li M Zhang Z Wang J Ri M. Licochalcone A inhibits proliferation and promotes apoptosis of colon cancer cell by targeting programmed cell death-ligand 1 via the NF-κB and Ras/Raf/MEK pathways. J Ethnopharmacol (2021) 273:113989. doi: 10.1016/j.jep.2021.113989 33677006

[77] Wu Y Zhu J Liu H Liu H. Licochalcone A improves the cognitive ability of mice by regulating T- and B-cell proliferation. Aging (Albany NY). (2021) 13(6):8895–915. doi: 10.18632/aging.202704 PMC8034954 33714945

[78] Motallebi M Bhia M Rajani HF Bhia I Tabarraei H Mohammadkhani N. Naringenin: A potential flavonoid phytochemical for cancer therapy. Life Sci (2022) 305:120752. doi: 10.1016/j.lfs.2022.120752 35779626

[79] Kawaguchi S Kawahara K Fujiwara Y. Naringenin potentiates anti-tumor immunity against oral cancer by inducing lymph node CD169-positive macrophage activation and cytotoxic T cell infiltration. Cancer Immunol Immunother (2022) 71(9):2127–39. doi: 10.1007/s00262-022-03149-w PMC9374624 35044489

[80] Wang L Zeng W Wang L Wang Z Yin X Qin Y. Naringenin enhances the antitumor effect of therapeutic V accines by promoting antigen cross-presentation. J Immunol (2020) 204(3):622–31. doi: 10.4049/jimmunol.1900278 31871020

[81] Qin L Jin L Lu L Lu X Zhang C Zhang F. Naringenin reduces lung metastasis in a breast cancer resection model. Protein Cell (2011) 2(6):507–16. doi: 10.1007/s13238-011-1056-8 PMC4875175 21748601

[82] Gong G Guan YY Zhang ZL Rahman K Wang SJ Zhou S. Isorhamnetin: A review of pharmacological effects. BioMed Pharmacother (2020) 128:110301. doi: 10.1016/j.biopha.2020.110301 32502837

[83] Paul A Das S Das J Samadder A Bishayee K Sadhukhan R. Diarylheptanoid-myricanone isolated from ethanolic extract of Myrica cerifera shows anticancer effects on HeLa and PC3 cell lines: signalling pathway and drug-DNA interaction. J Integr Med (2013) 11(6):405–15. doi: 10.3736/jintegrmed2013057 24299604

[84] Dai G Tong Y Chen X Ren Z Yang F. *In vitro* anticancer activity of myricanone in human lung adenocarcinoma A549 cells. Chemotherapy (2014) 60(2):81–7. doi: 10.1159/000371738 25720464

[85] Paul A Das J Das S Samadder A Khuda-Bukhsh AR. Anticancer potential of myricanone, a major bioactive component of Myrica cerifera: novel signaling cascade for accomplishing apoptosis. J Acupunct Meridian Stud (2013) 6(4):188–98. doi: 10.1016/j.jams.2013.05.003 23972241

[86] Peng F Du Q Peng C Wang N Tang H Xie X. A review: the pharmacology of isoliquiritigenin. Phytother Res Ptr (2015) 29:969–77. doi: 10.1002/ptr.5348 25907962

[87] Wang G Yu Y Wang Y-Z Yin P-H Xu K Zhang H. The effects and mechanisms of isoliquiritigenin loaded nanoliposomes regulated AMPK/mTOR mediated glycolysis in colorectal cancer. Artif Cells Nanomed Biotechnol (2020) 48:1231–49. doi: 10.1080/21691401.2020.1825092 32985258

[88] Wang KL Yu YC Hsia SM. Perspectives on the role of isoliquiritigenin in cancer. Cancers (Basel) (2021) 13(1):115. doi: 10.3390/cancers13010115 33401375 PMC7795842

[89] Tay KC Tan LT Chan CK Hong SL Chan KG Yap WH. Formononetin: A review of its anticancer potentials and mechanisms. Front Pharmacol (2019) 10:820. doi: 10.3389/fphar.2019.00820 31402861 PMC6676344

[90] Wang JY Jiang MW Li MY Zhang ZH Xing Y Ri M. Formononetin represses cervical tumorigenesis by interfering with the activation of PD-L1 through MYC and STAT3 downregulation. J Nutr Biochem (2022) 100:108899. doi: 10.1016/j.jnutbio.2021.108899 34748924

[91] Li D Wang G Jin G Yao K Zhao Z Bie L. Resveratrol suppresses colon cancer growth by targeting the AKT/STAT3 signaling pathway. Int J Mol Med (2018) 43(1):630–40. doi: 10.3892/ijmm.2018.3969 30387805

[92] Abu-Eid R Samara RN Ozbun L Abdalla MY Berzofsky JA Friedman KM. Selective inhibition of regulatory T cells by targeting the PI3KAkt pathway. CancerImmunol Res (2014) 2(11):1080–1089. doi: 10.1158/2326-6066.CIR-14-0095 PMC4221428 25080445

[93] Xu R Wu J Zhang X Zou X Li C Wang H. Modified Bu-zhong-yi-qi decoction synergies with 5 fluorouracile to inhibits gastric cancer progress via PD-1/PD- L1-dependent T cell immunization. Pharmacol Res (2020) 152:104623. doi: 10.1016/j.phrs.2019.104623 31899315

[94] Schaub FX Dhankani V Berger AC Trivedi M Richardson AB Shaw R. Pan- cancer alterations of the MYC oncogene and its proximal network across The Cancer Genome Atlas. Cell Syst (2018) 6:282–300.e2. doi: 10.1016/j.cels.2018.03.003 29596783 PMC5892207

[95] Kalkat M De Melo J Hickman KA Lourenco C Redel C Resetca D. MYC deregulation in primary human cancers. Genes (2017) 8:151. doi: 10.3390/genes8060151 28587062 PMC5485515

[96] Carroll PA Freie BW Mathsyaraja H Eisenman RN. The MYC transcription factor network: balancing metabolism, proliferation and oncogenesis. Front Med (2018) 12:412–25. doi: 10.1007/s11684-018-0650-z PMC7358075 30054853

[97] Casey SC Baylot V Felsher DW. The MYC oncogene is a global regulator of the immune response. Blood (2018) 131:2007–15. doi: 10.1182/blood-2017-11-742577 PMC5934797 29514782

[98] Casacuberta- Serra S Soucek L. Myc and Ras, the Bonnie and Clyde of immune evasion. Transl Cancer Res (2018) 7(Suppl. 4):S457–9. doi: 10.21037/tcr.2018.03.09 PMC6774775 31579305

[99] Yang C Liu Y Hu Y Fang L Huang Z Cui H. Myc inhibition tips the immune balance to promote antitumor immunity. Cell Mol Immunol (2022) 19(9):1030–41. doi: 10.1038/s41423-022-00898-7 PMC9424194 35962189

[100] Zhou M Liu X Li Z Huang Q Li F Li CY. Caspase-3 regulates the migration, invasion and metastasis of colon cancer cells. Int J Cancer (2018) 143(4):921–30. doi: 10.1002/ijc.31374 PMC6204286 29524226

[101] Dustin D Gu G Fuqua SAW. ESR1 mutations in breast cancer. Cancer (2019) 125(21):3714–28. doi: 10.1002/cncr.32345 PMC6788940 31318440

[102] Wang F Fu X Chen P Wu P Fan X Li N. SPSB1-mediated HnRNP A1 ubiquitylation regulates alternative splicing and cell migration in EGF signaling. Cell Res (2017) 27(4):540–58. doi: 10.1038/cr.2017.7 PMC5385621 28084329

[103] Hsu JL Hung MC. The role of HER2, EGFR, and other receptor tyrosine kinases in breast cancer. Cancer Metastasis Rev (2016) 35:575–88. doi: 10.1007/s10555-016-9649-6 PMC5215954 27913999

[104] Rotow J Bivona TG. Understanding and targeting resistance mechanisms in NSCLC. Nat Rev Cancer (2017) 17:637–58. doi: 10.1038/nrc.2017.84 29068003

[105] Roskoski R Jr. The ErbB/HER family of protein-tyrosine kinases and cancer. Pharm Res (2014) 79:34–74. doi: 10.1016/j.phrs.2013.11.002 24269963

[106] Madeddu C Donisi C Liscia N Lai E Scartozzi M Macciò A. EGFR-mutated non-small cell lung cancer and resistance to immunotherapy: role of the tumor microenvironment. Int J Mol Sci (2022) 23(12):6489. doi: 10.3390/ijms23126489 35742933 PMC9224267

[107] Rashid M Zadeh LR Baradaran B Molavi O Ghesmati Z Sabzichi M. Up-down regulation of HIF-1α in cancer progression. Gene (2021) 798:145796. doi: 10.1016/j.gene.2021.145796 34175393

[108] Barsoum IB Koti M Siemens DR Graham CH. Mechanisms of hypoxia-mediated immune escape in cancer. Cancer Res (2014) 74(24):7185–90. doi: 10.1158/0008-5472.CAN-14-2598 25344227

[109] Giatromanolaki A Koukourakis IM Balaska K Mitrakas AG Harris AL Koukourakis MI. Programmed death-1 receptor (PD-1) and PD-ligand-1 (PD-L1) expression in non-small cell lung cancer and the immune-suppressive effect of anaerobic glycolysis. Med Oncol (2019) 36(9):1–12. doi: 10.1007/s12032-019-1299-4 31342270

[110] Bailey CM Liu Y Liu M Du X Devenport M Zheng P. Targeting HIF-1α abrogates PD-L1-mediated immune evasion in tumor microenvironment but promotes tolerance in normal tissues. J Clin Invest (2022) 132(9):e150846. doi: 10.1172/JCI150846 35239514 PMC9057613

[111] Wiszniak S Schwarz Q. Exploring the intracrine functions of VEGF-A. Biomolecules (2021) 11(1):128. doi: 10.3390/biom11010128 33478167 PMC7835749

[112] Siveen KS Prabhu K Krishnankutty R Kuttikrishnan S Tsakou M Alali FQ. Vascular endothelial growth factor (VEGF) signaling in tumour vascularization: potential and challenges. Curr Vasc Pharmacol (2017) 15(4):339–51. doi: 10.2174/1570161115666170105124038 28056756

[113] Lapeyre-Prost A Terme M Pernot S Pointet AL Voron T Tartour E. Immunomodulatory activity of VEGF in cancer. Int Rev Cell Mol Biol (2017) 330:295–342. doi: 10.1016/bs.ircmb.2016.09.007 28215534

[114] Voron T Terme M. VEGF-A modulates expression of inhibitory checkpoints on CD8+ T cells in tumors. J Exp Med (2015) 212(2):139–48. doi: 10.1084/jem.20140559 PMC4322048 25601652

[115] Ozao-Choy J Chen SH. The novel role of tyrosine kinase inhibitor in the reversal of immune suppression and modulation of tumor microenvironment for immune-based cancer therapies. Cancer Res (2009) 69(6):2514–22. doi: 10.1158/0008-5472.CAN-08-4709 PMC4370269 19276342

[116] Meng Q Xia Y. c-Jun, at the crossroad of the signaling network. Protein Cell (2011) 2(11):889–98. doi: 10.1007/s13238-011-1113-3 PMC4875184 22180088

[117] Suto R Tominaga K Mizuguchi H Sasaki E Higuchi K Kim S. Dominant-negative mutant of c-Jun gene transfer: A novel therapeutic strategy for colorectal cancer. Gene Ther (2004) 11(2):187–93. doi: 10.1038/sj.gt.3302158 14712303

[118] Yao K Chen H Lee MH Li H Ma W Peng C. a natural inhibitor of c-Jun N-terminal kinase 1. Cancer Prev Res (Phila) (2014) 7(1):139–49. doi: 10.1158/1940-6207.CAPR-13-0117 24253317

[119] Jiang H Liu X Knolhoff BL Hegde S Lee KB Jiang H. Development of resistance to FAK inhibition in pancreatic cancer is linked to stromal depletion. Gut (2020) 69(1):122–32. doi: 10.1136/gutjnl-2018-317424 PMC7167297 31076405

[120] Rebe C Ghiringhelli F. STAT3, a master regulator of anti-tumor immune response. Cancers (Basel) (2019) 11(9):1280. doi: 10.3390/cancers11091280 31480382 PMC6770459

[121] Huynh J Chand A Gough D Ernst M. Therapeutically exploiting STAT3 activity in cancer - using tissue repair as a road map. Nat Rev Cancer (2019) 19:82–96. doi: 10.1038/s41568-018-0090-8 30578415

[122] Yu H Kortylewski M Pardoll D. Crosstalk between cancer and immune cells:role of STAT3 in the tumour microenvironment. Nat Rev Immunol (2007) 7:41–51. doi: 10.1038/nri1995 17186030

[123] Zou S Tong Q Liu B Huang W Tian Y Fu X. Targeting STAT3 in cancer immunotherapy. Mol Cancer (2020) 19(1):145. doi: 10.1186/s12943-020-01258-7 32972405 PMC7513516

[124] Ersahin T Tuncbag N Cetin-Atalay R. The PI3K/AKT/mTOR interactive pathway. Mol Biosyst (2015) 11(7):1946–54. doi: 10.1039/C5MB00101C 25924008

[125] Zhao R Song Y Wang Y Huang Y Li Z Cui Y. PD-1/PD-L1 blockade rescue exhausted CD8+ T cells in gastrointestinal stromal tumours via the PI3K/Akt/mTOR signalling pathway. Cell Proliferation (2019) 52(3):e12571. doi: 10.1111/cpr.12571 30714229 PMC6536456

[126] Gao Y Yang J Cai Y Fu S Zhang N Fu X. IFN-γ-mediated inhibition of lung cancer correlates with PD-L1 expression and is regulated by PI3K-AKT signaling. Int J Cancer (2018) 143(4):931–43. doi: 10.1002/ijc.31357 29516506

[127] De Henau O Rausch M Winkler D Campesato LF Liu C Cymerman DH. Overcoming resistance to checkpoint blockade therapy by targeting PI3Kgamma in myeloid cells. Nature (2016) 539:443–7. doi: 10.1038/nature20554 PMC5634331 27828943

[128] Srikanth S Gwack Y. Orai1-NFAT signalling pathway triggered by T cell receptor stimulation. Mol Cells (2013) 35(3):182–94. doi: 10.1007/s10059-013-0073-2 PMC3887911 23483280

[129] Huang J. Current developments of targeting the p53 signaling pathway for cancer treatment. Pharmacol Ther (2021) 220:107720. doi: 10.1016/j.pharmthera.2020.107720 33130194 PMC7969395

[130] Liu J Zhang C Wang J Hu W Feng Z. The Regulation of Ferroptosis by Tumor Suppressor p53 and its Pathway. Int J Mol Sci (2020) 21(21):8387. doi: 10.3390/ijms21218387 33182266 PMC7664917

[131] Jin J Lin G Huang H Xu D Yu H Ma X. Capsaicin mediates cell cycle arrest and apoptosis in human colon cancer cells via stabilizing and activating p53. Int J Biol Sci (2014) 10(3):285–95. doi: 10.7150/ijbs.7730 PMC3957084 24643130

[132] Li D Wang G Jin G Yao K Zhao Z Bie L. Resveratrol suppresses colon cancer growth by targeting the AKT/STAT3 signaling pathway. Int J Mol Med (2019) 43(1):630–40. doi: 10.3892/ijmm.2018.3969 30387805

[133] Zhang S Wang Y Sun Y Zhao G Wang J Liu L. Hinokiflavone, as a MDM2 inhibitor, activates p53 signaling pathway to induce apoptosis in human colon cancer HCT116 cells. Biochem Biophys Res Commun (2022) 594:93–100. doi: 10.1016/j.bbrc.2022.01.032 35078113

[134] Kim HJ Kim SK Kim BS Lee SH Park YS Park BK. Apoptotic effect of quercetin on HT-29 colon cancer cells via the AMPK signaling pathway. J Agric Food Chem (2010) 58(15):8643–50. doi: 10.1021/jf101510z 20681654

[135] Liu G Chen T Ding Z Wang Y Wei Y Wei X. Inhibition of FGF-FGFR and VEGF-VEGFR signalling in cancer treatment. Cell Prolif (2021) 54(4):e13009. doi: 10.1111/cpr.13009 33655556 PMC8016646

[136] de Almeida PE Mak J Hernandez G Jesudason R Herault A Javinal V. Anti-VEGF treatment enhances CD8^+^ T-cell antitumor activity by amplifying hypoxia. Cancer Immunol Res (2020) 8(6):806–18. doi: 10.1158/2326-6066.CIR-19-0360 32238381

[137] Adachi Y Kamiyama H Ichikawa K Fukushima S Ozawa Y Yamaguchi S. Inhibition of FGFR reactivates IFNγ Signaling in tumor cells to enhance the combined antitumor activity of lenvatinib with anti-PD-1 antibodies. Cancer Res (2022) 82(2):292–306. doi: 10.1158/0008-5472.CAN-20-2426 34753772 PMC9397636

[138] Han Y Fan X Fan L Wu Y Zhou Z Wang G. Liujunzi decoction exerts potent antitumor activity in oesophageal squamous cell carcinoma by inhibiting miR-34a/STAT3/IL-6R feedback loop, and modifies antitumor immunity. Phytomedicine (2023) 111:154672. doi: 10.1016/j.phymed.2023.154672 36701994

[139] Wang Y Tan J Hu P Pei Q Wen Y Ma W. Traditional Chinese medicine compound, Bu Sheng Hui Yang Fang, promotes the proliferation of lymphocytes in the immunosuppressed mice potentially by upregulating IL-4 signaling. BioMed Pharmacother (2021) 134:111107. doi: 10.1016/j.biopha.2020.111107 33341059

